# An l-fucose-responsive transcription factor cross-regulates the expression of a diverse array of carbohydrate-active enzymes in *Trichoderma reesei*

**DOI:** 10.1371/journal.pgen.1011815

**Published:** 2025-08-11

**Authors:** Qinqin Zhao, Liwei Gao, Nuo Xu, Xiuting Zhang, Yuqi Qin, Yinbo Qu, Guodong Liu

**Affiliations:** 1 State Key Laboratory of Microbial Technology, Shandong University, Qingdao, Shandong, China; 2 Tobacco Research Institute of Chinese Academy of Agricultural Sciences, Qingdao, Shandong, China; 3 National Glycoengineering Research Center, Shandong University, Qingdao, Shandong, China; University of Georgia, UNITED STATES OF AMERICA

## Abstract

l-Fucose is a universal capping component of biomolecules found throughout all domains of life. Although fungi are renowned for their role in biomass recycling, the mechanisms by which they process l-fucose remain largely unknown. In this study, we elucidate a l-fucose-responsive system in *Trichoderma reesei*, a model fungus for plant cell wall degradation. Central to this system is the transcription factor FUR1, which is indispensable for growth on l-fucose. FUR1 orchestrates the expression of l-fucose catabolic enzymes, including an l-fucose dehydrogenase that exhibits distant homology to counterparts in bacteria and mammals. Through RNA sequencing and biochemical assays, we demonstrate that FUR1 also governs the enzymatic liberation of l-fucose by upregulating extracellular α-l-fucosidases. Intriguingly, FUR1 mediates l-fucose-triggered expression of a broad spectrum of enzymes that target diverse glycosidic bonds (e.g., β-glucuronidic, α-galactosidic, and β-xylosidic linkages) within complex carbohydrates. Expression of a constitutively active FUR1 mutant unlocked the production of otherwise silent glycosidases, substantially boosting the hydrolytic capacity of the fungal secretome on orange peel. These findings offer the first molecular insight into l-fucose sensing and metabolism in fungi, and advance our understanding of the fungal regulatory network for coordinated expression of biomass-degrading enzymes.

## Author summary

l-Fucose, is a widely present constituent of polysaccharides, glycoproteins, and glycolipids in plants, animals and bacteria. l-Fucose frequently presents itself as a terminal monosaccharide, and plays critical roles in cell communication, immunity and host-microbe interactions. While fungi are well-known for breaking down various biomass molecules (e.g., lignocellulose) in nature, their metabolic and regulatory machineries for l-fucose utilization are enigmatic. Here, we examine the transcriptional response of *Trichoderma reesei* to l-fucose. We discovered that an orphan transcription factor activates the expression of l-fucose catabolic enzymes as well as extracellular α-l-fucosidases. Additionally, we found that this transcription factor also mediates the cross-induction by l-fucose of a wide range of enzymes that break down different types of glycosidic bonds. These findings provide the first understanding of how a fungus senses and metabolize l-fucose, and shed light on the complex regulatory systems that allow fungi to efficiently degrade biomass. This knowledge could help improve industrial fungal strains for the production of more efficient biomass-degrading enzymes.

## Introduction

Microbial degradation of carbohydrates plays an important role in global carbon cycling, animal nutrition, and the sustainable utilization of bioresources. Fungi contain an extraordinary diversity of metabolic genes, and are integral to the process of polysaccharide decomposition [1–3]. To date, the biochemical pathways for the utilization of various polysaccharides, such as cellulose, xylan, and pectin, have been elucidated in fungi, with a particular focus on species of *Aspergillus* and *Trichoderma*. Nevertheless, rational engineering of filamentous fungi for enhanced polysaccharide-egrading enzyme production is constrained by gaps in our understanding of the underlying regulatory mechanisms.

In fungal cells, the synthesis of polysaccharide-degrading enzymes and the subsequent catabolism of released sugars are often coordinately regulated by pathway-specific transcriptional activators, predominantly from the fungal-specific Zn_2_Cys_6_ family [4]. For instance, XYR1/XlnR, a conserved transcription factor in filamentous ascomycetes, activates the expression of multiple extracellular xylanolytic enzymes, d-xylose transporters, as well as several intracellular enzymes that channel d-xylose to the pentose phosphate pathway [4,5]. AraR, a paralog of XYR1/XlnR, has been shown to positively regulate the expression of arabinolytic and l-arabinose catabolic enzymes within the Eurotiales order [6]. These transcription factors have been harnessed as strategic targets for the genetic engineering of fungal strains to enhance extracellular enzyme production [7,8]. In certain instances, the genes encoding catabolic enzymes and their corresponding transcription factors are genomically co-localized in fungi [9].

l-Fucose (6-deoxy-l-galactose) is one of the most widely existing components of biomolecules in prokaryotic and eukaryotic organisms [10]. Often found as a terminal residue, l-fucose is a constituent of various polysaccharides (e.g., plant xyloglucan), oligosaccharides (e.g., human milk oligosaccharides), glycoproteins (e.g., intestinal mucins), and glycolipids (e.g., blood group-related glycolipids). It plays pivotal roles in numerous biological processes, including development, immunity, and tumorigenesis [11–13]. To utilize l-fucose, microorganisms express l-fucosidases, which liberate l-fucose from fucosylated molecules [14]. The following steps for l-fucose catabolism have been primarily characterized in bacteria, where it is processed through either phosphorylative or nonphosphorylative routes, ultimately being cleaved into C3 compounds that feed into the central carbon metabolism [15,16]. The ability to utilize l-fucose has been demonstrated to be crucial for the colonization of *Campylobacter jejuni* in the gut of its host [17]. The production of l-fucosidases and the utilization of l-fucose for cell growth have been reported in several fungal species [18,19]. Nonetheless, the metabolic pathway underpinning these processes, the regulatory mechanisms involved, and the cellular response to this sugar remain enigmatic.

The filamentous fungus *Trichoderma reesei* is extensively utilized in the industrial production of cellulases and hemicellulases. The genome of *T. reesei* harbors four putative α-l-fucosidase genes [20], indicating the presence of a potential l-fucose utilization system in this organism. In this study, we identified a transcription factor, designated FUR1 (l-fucose-responsive regulator 1), which is essential for l-fucose utilization in *T. reesei*. FUR1 regulates a suite of target genes, including those encoding enzymes involved in the nonphosphorylative catabolism of l-fucose, α-l-fucosidases, and a range of enzymes that act on various glycosidic bonds within biomass. Furthermore, we demonstrate that the expression of a constitutively active mutant of FUR1 alters the composition of the secretome, and enhances its efficiency in the saccharification of orange peel. These results provide the first molecular understanding of l-fucose utilization and the associated regulation system for coordinating complex carbohydrate degradation in fungi.

## Results

### Functional inference of FUR1 through genomic and genetic analyses

Our investigation initially focused on the functional characterization of the closest homologue (protein ID: 60282) of the xylanolytic/cellulolytic transcriptional regulator XYR1 in *T. reesei*. Using RNA-seq data, we refined the gene model, revealing that it encodes an 876-amino acid protein (S1 Data). This protein features a Zn_2_Cys_6_ DNA-binding domain (residues 33–94) and a fungal-specific transcription factor domain (residues 474–594). Sequence alignment with XYR1 demonstrated 43.8% similarity across the entire protein.

**Fig 1:**
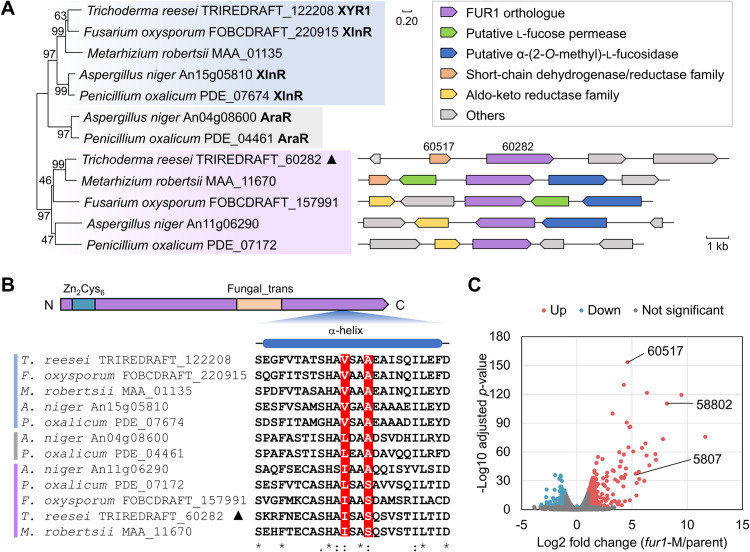
Linking the function of TRIREDRAFT_60282 (FUR1) to l-fucose utilization. **(A)** Phylogenetic analysis of FUR1 and its homologues in five fungal species. Functionally characterized proteins are highlighted in bold. Genome neighborhoods of the orthologues of TRIREDRAFT_60282 retrieved from the NCBI database are shown on the right. **(B)** Domain architecture of FUR1 and sequence alignment of its 746–770 region with homologous proteins. Residues corresponding to V821 and A824 in *T. reesei* XYR1 (TRIREDRAFT_122208) are highlighted in red. **(C)** Volcano plot of transcriptomic changes between *fur1*-M and parent strain. Samples were taken at 4 h after transferring mycelia to the carbon source-free medium.

The function of protein 60282 (hereafter designated FUR1) in l-fucose utilization was inferred by two approaches: (1) *Genomic context analysis.* Orthologues of FUR1 were identified across multiple fungal species within the Sordariomycetes and Eurotiomycetes classes. In *Fusarium oxysporum* and *Metarhizium robertsii*, the genomic neighborhoods of FUR1 orthologs contain genes encoding putative α-l-fucosidases and l-fucose permeases, suggesting conserved l-fucose-related gene clusters in these species (Fig 1A). Notably, genes annotated as NAD(P)-dependent oxidoreductases (InterPro IDs: IPR020471, IPR002347) are frequently co-localized with *fur1* [21]. (2) *Artificial activation of target genes.* A potential constitutively active mutant of *fur1* carrying double mutations (corresponding to I757F and S760V) was designed according to previous mutagenesis studies of XYR1/XlnR (Fig 1B) [22,23]. This mutant gene was overexpressed in *T. reesei* strain QMP, generating the engineered strain *fur1*-M. Comparison of the transcriptome of *fur1*-M in carbon source-free medium with that of the parent indicated that FUR1 predominantly functions as a transcriptional activator (Fig 1C, S2 Data). In *fur1*-M, a total of 319 genes were significantly upregulated, including all four putative α-l-fucosidase genes and a putative l-fucose permease gene (protein ID: 78833). Collectively, these results suggest FUR1’s role in regulating l-fucose release and assimilation. Interestingly, the gene upstream of *fur1* (protein ID: 60517, encoding a putative NAD(P)-dependent dehydrogenase) exhibited the most pronounced upregulation in *fur1*-M (Fig 1C).

### FUR1 is essential for l-fucose utilization and the expression of a novel l-fucose dehydrogenase

**Fig 2:**
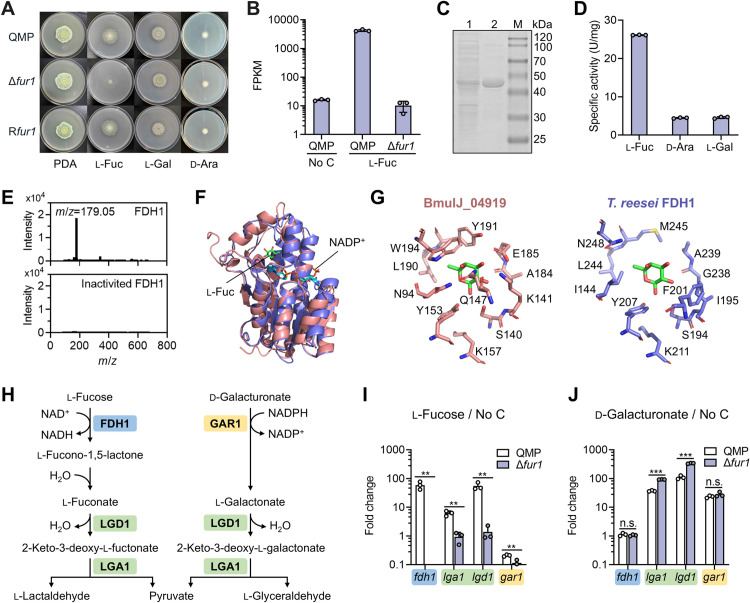
Regulation of l-fucose catabolic genes by FUR1. **(A)** The effect of *fur1* deletion on the growth of *T. reesei*. Strains were cultured on PDA or minimal medium with 0.5% (w/v) l-fucose, l-galactose and d-arabinose as the carbon source, respectively, for 5 days. The parental strain QMP was included as a control. **(B)** Transcript abundances of TRIREDRAFT_60517 after transferring mycelia to carbon source-free medium (No C) or minimal medium with 0.5% (w/v) l-fucose as the carbon source followed by cultivation for 4 h. **(C)** SDS-PAGE analysis of FDH1 recombinantly expressed in *E. coli*. Lane 1, soluble lysate; Lane 2, purified protein; M, molecular weight marker. **(D)** Specific activities of FDH1 on different sugars with NAD^+^ as a cofactor. **(E)** LC-ESI-MS analysis of reaction products from FDH1-catalyzed l-fucose oxidation. Mass spectra at 12.4 min elution are shown. Boiled FDH1 (inactivated by heating for 5 min) served as the negative control. **(F)** Comparison of the structure of BmulJ_04919-NADP^+^-l-fucose complex (PDB: 4GVX; pink) and the predicted structure of *T. reesei* FDH1 (blue). **(G)** Active site comparison between BmulJ_04919 and FDH1, with the latter obtained by superimposing the predicted structure of FDH1 onto that of BmulJ_04919. **(H)** Proposed l-fucose catabolic pathway in *T. reesei*, compared with the previously reported d-galacturonate catabolic pathway. **(I, J)** RT-qPCR analysis of the genes for l-fucose and d-galacturonate catabolism. Mycelia precultured in glycerol medium were transferred to minimal medium with 0.5% (w/v) l-fucose or 0.5% d-galacturonic acid as the carbon source, or No C medium, and then cultivated for 4 h. ***, *P* < 0.001; **, *P* < 0.01; *, *P* < 0.05; n.s., not significant. Data represent mean ± SD from triplicate cultivations.

To elucidate FUR1’s role in l-fucose metabolism, we generated a *fur1* deletion mutant (Δ*fur1*). While Δ*fur1* exhibited wild-type growth on potato dextrose agar and 10 synthetic media containing diverse carbon sources, it showed severely impaired growth when l-fucose served as the sole carbon source (Figs 2A and S1). Growth restoration in the complemented strain R*fur1* confirmed the phenotype’s genetic specificity. Transcriptome profiling of Δ*fur1* cultured in l-fucose medium (vs. parental strain QMP) revealed 309 differentially expressed genes, with TRIREDRAFT_60517 displaying the most pronounced downregulation (S2 Data). Notably, l-fucose-induced expression of TRIREDRAFT_60517 was completely abolished in Δ*fur1* (Fig 2B). Considering that 1) the TRIREDRAFT_60517 gene is co-located with *fur1* in the genome and its expression is positively regulated by FUR1; 2) the nonphosphorylative catabolic pathway of l-fucose in bacteria starts with a dehydrogenation reaction, we hypothesize that the protein 60517 might be involved in the l-fucose utilization process in *T. reesei*.

The re-annotated TRIREDRAFT_60517 gene encodes a 302-amino acid protein (S1 Data). Heterologous expression in *Escherichia coli* yielded a soluble recombinant protein, which was purified to homogeneity (Fig 2C). Biochemical assays revealed that the enzyme catalyzes the oxidation of l-fucose in the presence of NAD^+^, with a specific activity of 26.2 U/mg (Fig 2D). Mass spectrometry identified the reaction product as l-fuconate, with a monoisotopic mass of 179.05 Da (Figs 2E and S2). The *K*_m_ for l-fucose was estimated to be 0.546 mM under the optimal condition (pH 9.0 and 30°C; S3 Fig). No activity was detected when 0.5 mM NADP^+^ was used as a cofactor, suggesting that TRIREDRAFT_60517 is a NAD^+^-dependent l-fucose dehydrogenase, which was hereafter named FDH1.

Substrate specificity screening against 12 additional monosaccharides (d-fucose, d-xylose, l-xylose, d-arabinose, l-arabinose, d-galactose, l-galactose, d-ribose, d-glucose, d-mannose, d-fructose, l-rhamnose) revealed FDH1’s activity toward d-arabinose and l-galactose (17.5% relative to l-fucose; Fig 2D). The latter two sugars share the same hydroxyl group stereo-configurations at C2, C3 and C4 positions with l-fucose, a structural homology consistent with substrate preferences reported for prokaryotic and mammalian l-fucose dehydrogenases [24,25]. Nevertheless, the growth of *T. reesei* on d-arabinose and l-galactose remained unaffected in the Δ*fur1* mutant (Fig 2A), indicating the existence of FUR1-independent metabolic pathways for these substrates.

We then compared the predicted structure of FDH1 with the experimentally resolved structure of l-fucose dehydrogenase BmulJ_04919 from the bacterium *Burkholderia multivorans* (PDB: 4GVX, complexed with NADP^+^ and l-fucose). Despite sharing only 24.1% sequence identity, both enzymes adopt the canonical Rossmann fold characteristic of the short-chain dehydrogenase/reductase (SDR) family and exhibit significant structural overlap (Fig 2F). The critical catalytic residues (Y153, K157 and S140 in BmulJ_04919) are fully conserved in FDH1. However, divergent amino acid substitutions occur at positions coordinating the C2, C3, and C4 hydroxyl groups of l-fucose (Figs 2G and S4), suggesting distinct substrate recognition strategies. The basic residues R39 and H40 in BmulJ_04919—critical for NADP^+^ binding via 2′-phosphate interactions—are replaced by V88 and D89 in FDH1, explaining its strict NAD^+^ dependence. Also noted is that FDH1 has only 22.4% sequence identity with the recently identified l-fucose dehydrogenase HSD17B14 in human [24].

### FUR1 regulates more enzymes in the l-fucose catabolic pathway

The bacterial catabolism of l-fuconate typically proceeds via a dehydratase-mediated step, followed by either aldolase cleavage or tandem dehydrogenase-hydrolase reactions [16]. Previously, a l-galactonate dehydratase LGD1 essential for d-galacturonate metabolism in *T. reesei* (Fig 2H) was reported to be also active on d-arabonate [26]. LGD1 is possibly active on l-fuconate (6-deoxy-l-galactonate), which is structurally more similar to l-galactonate than d-arabonate. This hypothesis is partially supported by that the orthologue of LGD1 in *Aspergillus niger* (76% sequence identity), GaaB, demonstrates 10-fold higher activity on l-fuconate than its canonical substrate l-galactonate [27]. For the next step conversion, the 2-keto-3-deoxy-l-galactonate aldolase LGA1 in d-galacturonate metabolism has been reported to be actually much more active on 2-keto-3-deoxy-l-fuconate [16,28]. Based on these findings, we propose a metabolic pathway of l-fucose parallel with that of d-galacturonate in *T. reesei* (Fig 2H): (1) l-fucose is oxidized to l-fuconate via FDH1 and the followed spontaneous or enzymatic lactone hydrolysis; (2) l-fuconate undergoes dehydration by LGD1 to form 2-keto-3-deoxy-l-fuconate; (3) this intermediate is cleaved by LGA1 into l-lactaldehyde and pyruvate. Quantitative reverse transcription PCR (RT-qPCR) analysis confirmed l-fucose-dependent induction of *fdh1*, *lgd1*, and *lga1* in wild-type QMP, with complete abolition of this induction in Δ*fur1* (Fig 2I). Intriguingly, *lgd1* and *lga1*, but not *fdh1*, were also induced by d-galacturonate through a FUR1-independent mechanism (Fig 2J), while the d-galacturonate-specific gene *gar1* remained unresponsive to l-fucose. These results delineate a dual regulatory module governing l-fucose and d-galacturonate metabolism, involving FUR1-dependent transcriptional control for l-fucose catabolism and an unidentified d-galacturonate-responsive transcription factor.

### FUR1 activates the expression of α-l-fucosidases

**Fig 3:**
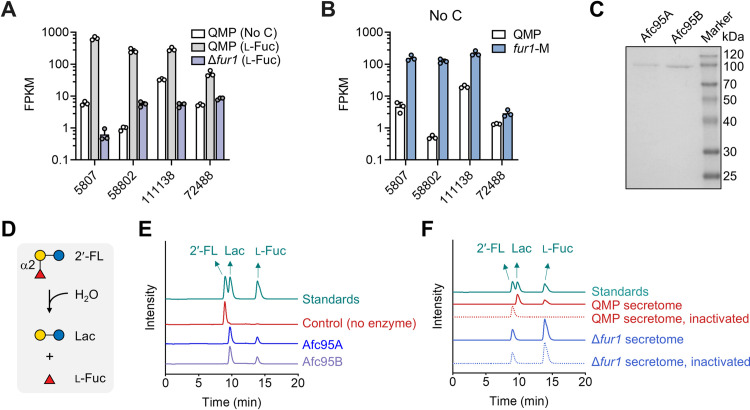
The role of FUR1 in α-l-fucosidase expression. **(A, B)** Transcript abundances of putative α-l-fucosidases after transferring mycelia to No C medium or minimal medium with 0.5% (w/v) l-fucose as the carbon source followed by cultivation for 4 h. Data represent mean ± SD from triplicate cultivations. **(C)** SDS-PAGE analysis of recombinant Afc95A and Afc95B secreted by *P. pastoris*. **(D)** The reaction of 2’-FL hydrolysis mediated by α-1,2-l-fucosidases. **(E, F)** HPLC analysis of 2′-FL hydrolysis products. For Afc95A and Afc95B, purified enzymes were used. For secretomes, culture supernatants of strains with mycelia transferred to minimal medium with 0.5% (w/v) l-fucose as the carbon source and then cultivated for 90 h were used. Boiled crude enzymes (heat-inactivated) served as negative controls. Detection of l-fucose in Δ*fur1* secretome is due to the inability of this strain to utilize l-fucose and thereby its accumulation in the culture.

Eukaryotic enzymes responsible for fucosidic bond cleavage are predominantly classified into glycoside hydrolase (GH) family 95 in the Carbohydrate-Active Enzymes database (www.cazy.org) [14,29]. Four genes encoding GH95 enzymes (protein IDs: 5807, 58802, 111138 and 72488) are annotated in *T. reesei*. The transcription levels of all four genes were induced by l-fucose in a *fur1*-dependent manner (Fig 3A), and elevated in the *fur1*-M strain in carbon source-free medium (Fig 3B). We selected 5807 and 58802, which exhibiting the strongest l-fucose-responsive induction among the four genes, for heterologous expression in *Pichia pastoris* (Fig 3C). Both recombinant enzymes hydrolyzed the α-1,2-fucosylgalactose linkage in 2’-fucosyllactose (2’-FL) (Fig 3D and 3E). Therefore, 5807 and 58802 were named Afc95A and Afc95B, respectively. Notably, neither enzyme displayed activity toward *p*-nitrophenyl α-l-fucopyranoside, aligning with substrate specificity patterns reported for two characterized GH95 fucosidases [30,31]. Consistent with these findings, crude extracellular extracts from l-fucose-grown QMP efficiently cleaved 2’-FL into lactose and l-fucose, whereas this activity was absent in the Δ*fur1* secretome under identical conditions (Fig 3F). These results establish FUR1 as the master transcriptional regulator of all secreted α-1,2-l-fucosidases in *T. reesei*.

### FUR1 mediates l-fucose-induced expression of multiple biomass-degrading enzymes

**Fig 4:**
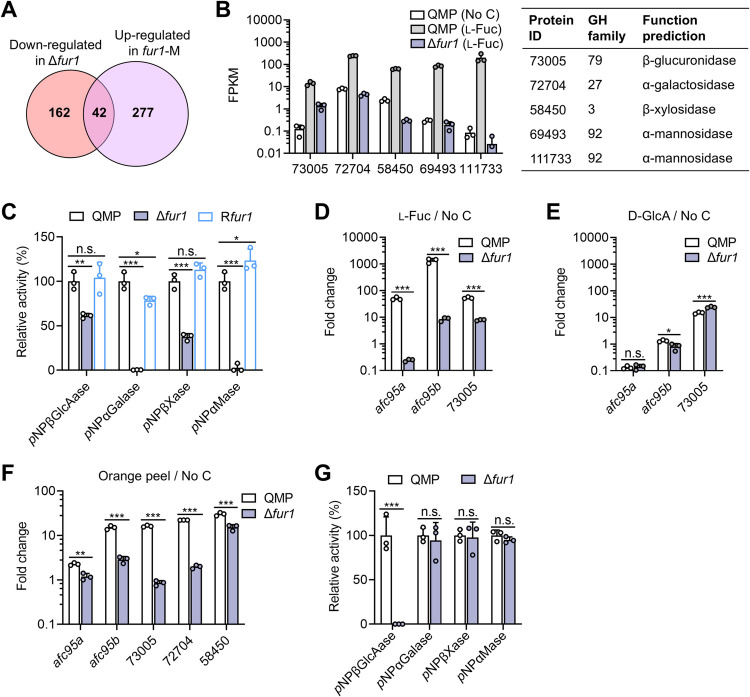
FUR1 regulates the expression of multiple biomass-degrading enzymes. **(A)** Overlap analysis of genes upregulated in *fur1*-M (No C medium) and downregulated in Δ*fur1* (l-fucose medium), defining a “core regulon”. **(B)** FPKM values of five genes encoding biomass-degrading enzymes in Δ*fur1* and parantal strain QMP. **(C)** Relative activities of extracellular β-glucuronidase, α-galactosidase, β-xylosidase and α-mannosidase. QMP (set as 100%) and mutant strains were cultured in minimal medium with 0.5% (w/v) l-fucose as the carbon source for 60 h. **(D–F)** Induced expression of biomass-degrading enzyme genes by various carbon sources in QMP and Δ*fur1*. Mycelia were transferred to minimal medium containing 0.5% l-fucose **(D)**, 0.5% d-glucuronic acid **(E)** or 0.5% orange peel **(F)** and then cultivated for 4 h. The fold changes of transcript abundances relative to those in No C medium are shown. **(G)** Relative activities of biomass-degrading enzyme of QMP (set as 100%) and Δ*fur1* in 2.0% (w/v) orange peel medium after cultivation of 120 h. ***, *P* < 0.001; **, *P* < 0.01; *, *P* < 0.05; n.s., not significant. Data represent mean ± SD from triplicate cultivations.

The above transcriptomic studies identified 319 genes significantly upregulated in *fur1*-M strain in carbon source-free medium and 204 genes downregulated in Δ*fur1* under the l-fucose inducing condition, compared to the parent strain. Overlapping of the two datasets revealed 42 genes as the “core regulon” of FUR1 (Fig 4A and S2 Data). In addition to *fdh1* and α-l-fucosidase genes, this regulon encompasses diverse biomass-degrading enzymes, including putative β-xylosidases, α-galactosidases, and α-mannosidases. These genes exhibited the hallmark expression profile of FUR1 targets: l-fucose-inducible and FUR1-dependent activation (Fig 4B). Functional validation through extracellular enzyme activity assays in l-fucose medium confirmed the regulatory role of FUR1 (Fig 4C).

Given FUR1’s activation of genes unrelated to l-fucose catabolism, we investigated whether this regulation strictly requires l-fucose as an inducer. For TRIREDRAFT_73005, a FUR1 target gene encoding a putative β-glucuronidase, RT-qPCR confirmed FUR1-dependent induction under the l-fucose condition (Fig 4D). While d-glucuronate (a β-glucuronidase reaction product) also induced TRIREDRAFT_73005 expression, this induction occurred independently of FUR1 (Fig 4E). Combined with prior observations of FUR1-independent d-galacturonate-responsive genes (Fig 2J), these findings indicate that FUR1’s regulatory input is specifically tuned to l-fucose, even as it governs a diverse array of downstream targets.

In natural ecosystems, l-fucose predominantly exists as glycoconjugates rather than free monomers, limiting microbial exposure to pure l-fucose as a carbon source. To assess FUR1’s regulatory role in complex biomass utilization, we employed orange peel, a natural substrate containing l-fucose alongside at least seven other monosaccharides (e.g., d-galacturonic acid, d-glucose) [32]. Transcript analysis revealed significantly reduced expression of six FUR1 target genes in Δ*fur1* versus QMP during orange peel cultivation (Fig 4F). Notably, the putative β-xylosidase gene TRIREDRAFT_58450 retained orange peel-induced expression relative to carbon starvation, albeit at reduced levels in Δ*fur1*. Consistent with impaired induction of TRIREDRAFT_73005, extracellular β-glucuronidase activity in the orange peel medium was markedly diminished in Δ*fur1* (Fig 4G). In contrast, β-xylosidase and α-galactosidase activities remained unaffected by *fur1* deletion, likely due to compensatory induction of isozymes by alternative sugars in orange peel. These findings position FUR1 as a collaborative regulator that acts together with substrate-specific transcription factors to orchestrate polysaccharide degradation in natural environments.

### Sequence determinants underlying functional divergence between FUR1 and XYR1

**Fig 5:**
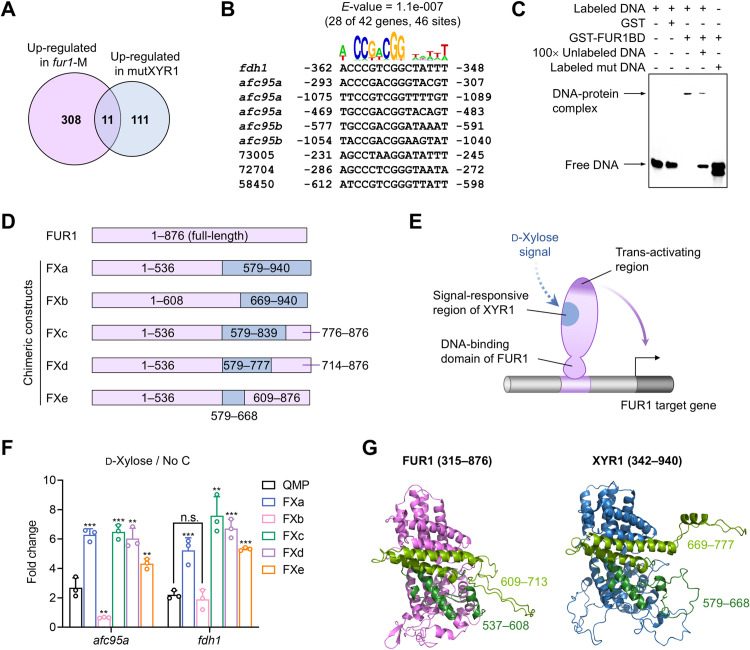
Differences in DNA-binding and signal response between FUR1 and XYR1. **(A)** Comparison of genes upregulated in *fur1*-M strain (No C medium) in this study with the genes upregulated in XYR1-activated mutant in literature [33]. **(B)** Sequence logo representation of the consensus sequence discovered in the upstream sequences of 42 FUR1 “core regulon” genes. Examples of nine sequences are shown. Numbers indicate distance from the start codon. **(C)** EMSA assay showing the binding of FUR1 to the DNA fragment containing the upstream sequence of *fdh1* shown in panel B. GST-FUR1BD, glutathione S-transferase tagged DNA-binding domain of FUR1. **(D)** Schematic illustration of the chimeric transcription factors expressed from the *fur1* locus in the genome-edited strains. Sequences derived from FUR1 and XYR1 are shown in purple and blue color, respectively. **(E)** The hypothesis that a chimeric FUR1 mutant carrying the signal-responsive region of XYR1 would activate the expression of FUR1 targets in the presence of d-xylose. **(F)** RT-qPCR analysis of *afc95a* and *fdh1* expression in response to d-xylose. Mycelia precultured in glycerol medium were transferred to minimal medium with 0.5% (w/v) d-xylose as the carbon source, or No C medium, and then cultivated for 18 h. The statistical significance of the difference between QMP and each mutant strain is shown. ***, *P* < 0.001; **, *P* < 0.01; *, *P* < 0.05; n.s., not significant. Data represent mean ± SD from triplicate cultivations. **(G)** Predicted structural localization of the signal-discriminatory region (green and light green color) in FUR1/XYR1. N-terminal DNA-binding domains are omitted for clarity.

To distinguish the regulatory functions of FUR1 from its homologue XYR1, we compared transcriptomic datasets from *T. reesei* strains expressing constitutively active mutants of these regulators: 319 genes upregulated in *fur1*-M (this study) versus 122 genes upregulated in mutXYR1 (reported by Sveholm et al. [33]). Only 11 genes overlapped between these sets (Fig 5A), including *xyn4* (TRIREDRAFT_111849, encoding a xylanase) and TRIREDRAFT_58450 (putative β-xylosidase gene), demonstrating distinct regulatory networks governed by FUR1 and XYR1. Motif analysis of 1.2-kb upstream regions from all 319 FUR1-activated genes revealed no significant consensus sequences. However, the 42 “core regulon” genes exhibited strong enrichment for the heptameric motif 5′-CCGACGG-3′ flanked by AT-rich sequences (Fig 5B). Electrophoretic mobility shift assay (EMSA) confirmed FUR1’s specific binding to this motif upstream of *fdh1* (Fig 5C). Crucially, mutating the motif to XYR1’s canonical binding sequence (5′-GGCTAAA-3′) [34] abolished DNA-protein interaction, establishing 5′-CCGACGG-3′ as the FUR1-specific *cis*-regulatory element.

Generally, transcriptional activators possess distinct domains or surfaces for DNA-binding, trans-activating and signal-responsive functions. In d-xylose medium, where the regulatory activity of XYR1 is physiologically activated [5], extracellular β-xylosidase, β-glucuronidase and α-galactosidase activities remained unaffected by *fur1* deletion (S5 Fig). This result suggests that FUR1 and XYR1 respond to distinct inducers. Given their full-length sequence similarity, we hypothesized that the structural differences in a certain region of FUR1/XYR1 mediate their signal-specific regulation. To test this, we engineered chimeric FUR1 variants by replacing defined segments of *fur1* with homologous *xyr1* sequences via CRISPR/Cas9 (Figs 5D, S6 and S7). Theoretically, such hybrids could activate FUR1 targets in d-xylose medium if the signal-sensing domain is swapped (Fig 5E). All the mutant strains failed to grow on l-fucose (S8 Fig), confirming disruption of native l-fucose signaling. Strikingly, replacing the C-terminal 340 amino acids (residues 537–876) of FUR1 with the corresponding XYR1 region (residues 579–777) enabled d-xylose-responsive induction of *afc95A* and *fdh1* (Fig 5F). Fine-mapping narrowed the critical region to residues 579–777 of XYR1 (equivalent to FUR1 537–713), with residues 579–668 being essential. Structural predictions indicate this discriminatory region forms surface-exposed α-helices and loops (Fig 5G), though its mechanism of signal recognition remains unresolved.

### FUR1 engineering enhances secretory protein diversity and biomass-degradation efficiency

**Fig 6:**
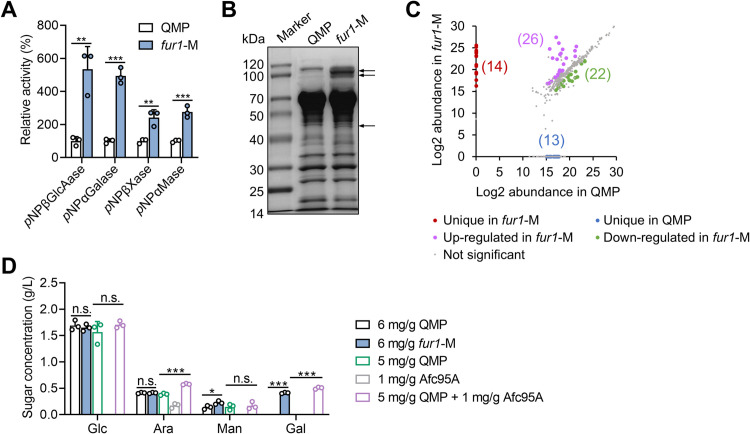
Constitutive activation of FUR1 improves biomass-degrading efficiency of the secretome. The culture supernatants of QMP and *fur1-*M cultivated in the medium with 2.0% (w/v) microcrystalline cellulose as carbon source for 120 h were collected for comparisons. **(A)** Relative activities of carbohydrate-active enzymes, with those of QMP set as 100%. **(B)** SDS-PAGE analysis of culture supernatants of equal volume (16 μl). Protein bands uniquely detected in *fur1-*M enzymes are indicated by arrows. **(C)** Comparison of protein abundances between the secretomes of QMP and *fur1-*M. The numbers of proteins in each group are shown in parentheses. **(D)** The concentrations of sugars released from orange peel by different enzyme mixtures. The dosages of enzymes (mg protein per gram of substrate) are shown. HPLC analysis failed to detect l-fucose in the hydrolysates, likely because of its low abundance in orange peel. ***, *P* < 0.001; **, *P* < 0.01; *, *P* < 0.05; n.s., not significant. Data represent mean ± SD from triplicate cultivations (panels **A** and **C**) or triplicate enzymatic reactions (panel D).

The positive regulation of multiple biomass-degrading enzymes by FUR1 highlights its potential as a genetic engineering target for optimizing secretome composition. To validate this, we compared extracellular proteins produced by the *fur1*-M mutant and parental strain QMP. In l-fucose medium, the engineering of FUR1 significantly boosted the induction of multiple extracellular hydrolytic enzymes (S9 Fig). In the medium with microcrystalline cellulose as carbon source, a standard condition for lignocellulolytic enzyme production by *T. reesei*, although total protein concentrations showed no significant differences, extracellular activities of β-glucuronidase, α-galactosidase, β-xylosidase, and α-mannosidase in *fur1*-M were 0.7- to 3.8-fold higher than those in QMP (Fig 6A). SDS-PAGE analysis revealed additional protein components secreted into the medium by *fur1*-M (Fig 6B). Quantitative proteomic profiling of extracellular proteins identified 412 detected species, with 26 significantly upregulated and 22 downregulated in *fur1-M* versus QMP (Fig 6C and S1 Table). Strikingly, 14 proteins, including the α-l-fucosidases Afc95A and Afc95B, were exclusively detected in *fur1*-M.

The secretomes of both strains were then evaluated for their efficiency in orange peel degradation. After 72 hours of enzymatic hydrolysis, extracellular enzymes from *fur1*-M released 0.41 g/L of galactose, whereas no galactose was detected in samples treated with QMP enzymes (Fig 6D). Additionally, *fur1*-M enzymes produced 64.2% more mannose than QMP at equivalent protein concentrations. When recombinant Afc95A was supplemented into QMP’s extracellular enzymes, 0.50 g/L of galactose was detected in the hydrolysate, and the concentration of arabinose increased by 49.7% (Fig 6D). Since Afc95A alone did not liberate galactose from orange peel, the galactose production by *fur1*-M enzymes was attributed to the removal of “capping” l-fucose residues via α-l-fucosidases enriched in its secretome.

## Discussion

The molecular mechanisms underlying fungal l-fucose utilization have remained enigmatic. Our study reveals FUR1 as the pivotal transcription factor essential for l-fucose catabolism in *T. reesei*. We further propose an FUR1-regulated metabolic pathway initiated by FDH1-mediated oxidation of l-fucose—a mechanism analogous to the Entner-Doudoroff pathway. Strikingly, this pathway is conserved across domains of life, with functional analogs documented in bacteria, archaea, and human [17,24,35]. Nevertheless, the sequences of l-fucose dehydrogenases from distinct lineages are distantly related from each other, suggesting convergent evolution of this catalytic function through independent molecular trajectories. Notably, the pathway exhibits modular plasticity through enzyme sharing: the dehydratase (LGD1) and aldolase (LGA1) catalyzing subsequent steps are functionally promiscuous, participating in both l-fucose and d-galacturonate metabolism (and potentially d-arabinose catabolism) [36]. This metabolic “toolkit sharing” strategy likely enhances substrate flexibility while minimizing genomic redundancy.

FUR1 also mediates the feed-back transcriptional activation of GH95 α-l-fucosidases by l-fucose. Both Afc95A and Afc95B characterized in this study are predicted to be secreted proteins. They may facilitate *T. reesei*’s saprophytic lifestyle by enabling the utilization of fucosylated substrates in decaying organic matter. Specifically, these enzymes are supposed to act on α-1,2-fucosyl linkages prevalent in plant biomass components (e.g., xyloglucan, rhamnogalacturonan, and arabinogalactan proteins) [37,38] and mammalian *O*-glycosylated proteins [39,40]. Phylogenetic analysis reveals that FUR1 orthologues are encoded in some *Fusarium* and *Metarhizium* genomes, occasionally co-localized with α-l-fucosidase genes (Fig 1A), suggesting an evolutionarily conserved regulatory mechanism for l-fucose-responsive induction of α-l-fucosidases [41]. Whether this glycan degradation system is involved in the interaction of these pathogenic fungi with their hosts remains to be investigated.

One striking finding of this study is l-fucose’s ability to induce enzymes targeting non-fucosylated substrates (Fig 4). The products of these enzymes include glucuronic acid, galactose, xylose and mannose, which are commonly associated with fucosylated glycans [42–44]. Notably, FUR1 activates several genes encoding uncharacterized extracellular hydrolases (e.g., TRIREDRAFT_109392, TRIREDRAFT_105288) in response to l-fucose, suggesting broader induction of biomass-degrading machinery in *T. reesei*. We propose that l-fucose functions as a “signal” of environmental complex glycans for *T. reesei*. When terminal l-fucose residues are liberated from heteropolysaccharides or oligosaccharides, they may trigger rapid secretion of diverse hydrolases, enabling efficient deconstruction of complex glycans for nutrient acquisition. Interestingly, this signaling role of l-fucose was also reported in enterohaemorrhagic *E. coli*, where a two-component system senses l-fucose in intestine and modulates the expression of virulence genes [45].

The functional characterization of FUR1 expands the fungal biomass-degradation regulatory network by introducing a novel transcriptional regulator. FUR1 joins XYR1/XlnR, AraR, and GalR (the latter being unique to *Aspergillus nidulans* for d-galactose utilization [46]) as members of a conserved gene family governing carbohydrate metabolism. Structural similarities among these transcription factors suggest they employ shared molecular mechanisms for sugar signal perception and transcriptional activation (Fig 5). This process likely involves regulated nuclear translocation, though the complete mechanistic framework awaits full elucidation [22]. The evolutionary conservation of FUR1 presents intriguing questions. While FUR1 orthologues are widely distributed across ascomycete fungi, species like *A. niger* lack l-fucose utilization capacity [27]. Notably, *A. niger* harbors the FUR1 ortholog An11g06290 genomically co-localized with a putative d-arabinose dehydrogenase gene (An11g06280), suggesting functional repurposing to regulate d-arabinose metabolism, a structural analogue of l-fucose.

The functional discovery of FUR1 holds significant promise for industrial applications. Although l-fucose occurs at low abundance in most glycans, it serves as a protective capping moiety that impedes enzymatic degradation of adjacent glycosidic linkages [40,47]. While *T. reesei* encodes more α-l-fucosidase genes than many other biomass-degrading fungi (e.g., *Myceliophthora thermophila* and *A. niger*), these enzymes are minimally secreted under conventional lignocellulolytic enzyme production conditions [48,49]. Our findings demonstrate that FUR1 engineering enhances α-l-fucosidase production and broadens the hydrolytic enzyme repertoire in *T. reesei* secretome without media modification, achieving a significant increase in galactose yield from orange peel hydrolysis (Fig 6). This strategy of transcriptional regulator engineering merits validation in other industrial fungi to refine secretome composition for tailored biomass deconstruction.

**Fig 7:**
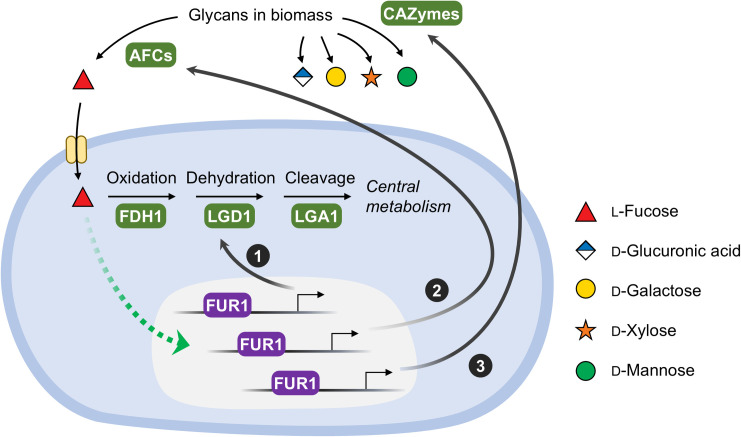
Coordinated regulatory network of l-fucose-responsive metabolism in *T. reesei.* In the presence of l-fucose, FUR1 activates the expression of three groups of enzymes associated with carbohydrate utilization: (1) l-fucose catabolic enzymes; (2) extracellular α-l-fucosidases (AFCs) for l-fucose release from glycans; (3) extracellular carbohydrate-active enzymes (CAZymes) targeting non-fucosyl linkages for synergistic biomass deconstruction. A dashed green arrow denotes unresolved signaling mechanisms for L-fucose perception and FUR1 activation.

In summary, this study elucidates a sophisticated regulatory framework through which *T. reesei* coordinates l-fucose utilization with multi-enzyme biomass deconstruction (Fig 7). The transcription factor FUR1 senses environmental l-fucose and subsequently activates not only intracellular L-fucose catabolic pathways but secretory systems for liberating l-fucose and associated monosaccharides from heteroglycans. This cross-induction mechanism reduces the cost of cell to regulate the utilization of complex carbohydrates. These findings provide new insights into the bio-recycling capacity of fungi, and suggest potential strategies for rational design of industrial strains and enzyme cocktails for highly-efficient biomass degradation.

## Materials and methods

### Construction of *T. reesei* mutants

*T. reesei* QMP, a uracil auxotrophic strain derived from the strain QM9414 (ATCC 26921) through deleting the *pyr4* gene, was used as the parent for strain construction. To construct *fur1* deletion strain Δ*fur1*, the flanking regions of *fur1* gene were amplified by PCR respectively, and fused with the *A. nidulans pyrG* (encoding orotidine-5′-phosphate decarboxylase) as a selection marker gene by overlap extension PCR, generating the knock-out cassette for homologous recombination in QMP. For gene complementation, the *fur1* gene along with its 1.6-kb upstream and 0.5-kb downstream sequences was fused with hygromycin B resistance gene (*hph*) and transformed into Δ*fur1*. To construct strain *fur1-*M, the sequence encoding point mutant FUR1^I757F, S760V^ was obtained by overlap extension PCR incorporating nucleotide changes into the overlapping primers, and then fused with the promoter of *cdna1* gene, terminator of *A. nidulans trpC gene,* and selectable marker gene *pyrG*, to generate the expression cassette P_*cdna1*_-*fur1*^*I757F, S760V*^-T_*AntrpC*_-*pyrG*. The primers used for strain construction are listed in S2 Table.

For the construction of FXa–e strains, donor DNA fragments were integrated at the *fur1* locus by homologous recombination using the CRISPR/Cas9-based genome editing method. The donor DNAs consisted of partial sequence of *xyr1* flanked by homologous arms from the *fur1* locus. For each editing experiment, two single guide RNAs (sgRNAs) were used to introduce double-strand breaks at the *fur1* gene. Plasmids containing *Streptococcus pyogenes cas9* expression cassette, selection marker gene *A. nidulans pyrG*, autonomously replicating sequence AMA1, and sgRNA array cassette (composed of the following fragments in order: 5*S* rRNA promoter, first protospacer, sgRNA scaffold, tRNA^GLY^, second protospacer, sgRNA scaffold, terminator), were constructed using overlap extension PCR and the ClonExpress II One Step Cloning Kit (Vazyme, Nanjing, China) [50]. The donor DNA and corresponding plasmid for Cas9 and sgRNA expression were co-transformed into QMP strain, respectively. Protoplast-mediated transformation was performed as described by Penttila *et al* [51]. Transformants were purified by plate streaking, and the genotypes of strains obtained were identified by PCR and Sanger sequencing. The primers used for strain construction are listed in S3 Table.

### Cultivation of *T. reesei*

The strains were cultivated on potato dextrose agar (PDA) plates at 30°C for 5 days for conidiation. To test the growth on different carbon sources, 2 μL of conidial suspension (10^7^ per ml) was inoculated on the center of agar plates prepared by supplementing indicated carbon source to minimal medium, and cultivated at 30°C for 5 days. Triton X-100 was added into the solid medium at a final concentration of 0.05% (w/v). For liquid cultivation, fresh conidia were inoculated into minimal medium with 1.0% (w/v) glycerol as the carbon source with a final concentration of 10^6^ per ml, and incubated in a rotary shaker at 200 rpm at 30°C for 30 h. For RNA extraction and the preparation of crude enzymes for 2’-FL hydrolysis, the mycelia precultured in glycerol medium were harvested by filtration, and 0.2 g (wet weight) of mycelia was inoculated to 50 ml indicated medium for continued cultivation. For extracellular enzyme activity measurement, the culture in glycerol medium was directly inoculated to minimal medium supplemented with indicated carbon source with an inoculation ratio of 10% (v/v) for continued cultivation. The minimal medium contained (g/l): carbon source as indicated, (NH_4_)_2_SO_4_ 5.0, KH_2_PO_4_ 15.0, MgSO_4_·7H_2_O 0.6, CaCl_2_ 0.6, peptone 2.0, FeSO_4_·7H_2_O 0.005, MnSO_4_·H_2_O 0.0016, ZnSO_4_·7H_2_O 0.0014, and CoCl_2_·6H_2_O 0.002. For comparison of the enzyme activities, protein composition and biomass-degrading efficiencies of extracellular proteins between QMP and *fur1-*M, the culture in glycerol medium was inoculated to the following medium with an inoculation ratio of 10% (v/v) for enzyme production (g/l): microcrystalline cellulose 20, corn steep liquor 20.0, KH_2_PO_4_ 5.0, (NH_4_)_2_SO_4_ 2.0, MgSO_4_·7H_2_O 0.6, and CaCl_2_ 1.0.

### Heterologous expression and purification of proteins

For the expression of FDH1, the *fdh1* gene was codon-optimized according to the *E. coli* bias (S1 Data). The synthesized gene was inserted into the pET-32a(+) vector between the *Eco*RI and *Not*I sites, and the recombinant plasmid was transformed into *E. coli* BL21(DE3). The transformant was grown in Luria–Bertani medium containing 100 μg/ml ampicillin at 37°C to an OD_600_ of 0.5. Then, FDH1 expression was induced by the addition of 0.5 mM isopropyl-β-d-thiogalactopyranoside (IPTG) and cultivation at 16°C for 12 h. Cells were harvested and lysed by sonication. The FDH1 protein with a N-terminal 6 × histidine tag was purified with a His Trap FF Column (Cytiva, USA), and desalted by dialysis in PBS. The DNA-binding domain of FUR1 (amino acids 32–94) was expressed in *E. coli* using the same method, except that the coding sequence was inserted into pGEX-4T-1 vector between the *Eco*RI and *Not*I sites, and 0.2 mM IPTG was used for induction. The GST-FUR1BD protein was purified using Glutathione Beads (Smart-Lifesciences, China), and desalted by dialysis in PBS.

For the expression of Afc95A and Afc95B, the sequences encoding the mature proteins were amplified from the cDNA of QMP and cloned into pPIC9K vector between the *Eco*RI and *Not*I sites. Six codons for histidine were added before the stop codon during the amplification process. The recombinant plasmids extracted from *E. coli* were linearized with *Bgl*II (for *afc95A*) or *Sac*I (for *afc95B*), and then electroporated into *P. pastoris* GS115. The recombinant *P. pastoris* strains were grown in YPD medium at 30°C for 24 h and then inoculated into BMGY medium for cultivation at 30°C for 18 h. The cells were then transferred into BMMY medium to an OD_600_ of 1.0. and cultivated at 30°C for 120 h. Methanol was added every 24 h to a final concentration of 1.0% (v/v) to induce protein expression. Target enzymes were purified from culture supernatants with His Trap FF Column (Cytiva, USA), and exchanged to 0.1 M citrate-phosphate buffer (pH 5.0) using Nanosep 10K centrifugal devices (Pall, USA). The YPD medium contained (g/l): glucose 20.0, peptone 20.0, yeast extract 10.0. The BMGY medium contained (g/l): glycerol 10.0, peptone 20.0, yeast extract 10.0, yeast nitrogen base 3.4, (NH_4_)_2_SO_4_ 10.0, KH_2_PO_4_ 11.8, K_2_HPO_4_ 3.0, and biotin 0.0004. The composition of BMMY medium was the same as that of BMGY except that glycerol was replaced by methanol (1.0%, v/v). The primers used for heterologous gene expression are listed in S4 Table. The concentration of proteins was measured using the Modified Bradford reagent (Sangon, Shanghai, China).

### Enzyme assays and product analysis

The l-fucose dehydrogenase activity was determined using a previously reported method with slight modifications [24]. Briefly, the activity was assayed at 30°C by monitoring the linear increase in absorbance at 340 nm as NAD^+^ was reduced to NADH in 10 min. The reaction mixture contained 50 mM Tris-HCl (pH 9.0), 0.5 mM NAD^+^, 6 mM l-fucose and recombinant FDH1. One unit of activity was defined as the amount of enzyme required to produce 1 μmol of NADH per minute. The activity toward NADP^+^ was measured using the same method, with 0.5 mM NADP^+^ replacing 0.5 mM NAD^+^. For kinetics study, the initial reaction rates of FDH1 were determined using l-fucose over a concentration range of 0.083 to 10 mM. A Hill equation was fitted to the data to estimate *K*_m_ and *V*_max_ using OriginPro 2021 (OriginLab, USA). To determine the product of the FDH1-catalyzed reaction, the reaction solution containing 20 mM l-fucose, 2 mM NAD^+^, 150 mM NaCl, 0.13 mg/ml purified FDH1 in 50 mM phosphate buffer (pH 9.0) was incubated at 30°C for 1 h. The sample was subjected to HPLC-ESI-MS analysis using an Aminex HPX-87H column (Bio-Rad, USA) with 0.1% (v/v) formic acid as mobile phase at a flow rate of 0.5 ml/min. High-resolution Q-TOF mass spectrometry (impactHD, Bruker, Germany) was performed in the negative electrospray ionization mode. MS data were processed using DataAnalysis 4.2 (Bruker, Germany).

The hydrolytic activity of purified α-l-fucosidases was investigated by incubating 0.5 mg 2′-FL (Aladdin, China) with 5 μg enzyme with a reaction volume of 600 μL in 0.1 M citrate-phosphate buffer (pH 5.0) at 50°C for 4 h. A similar reaction system with a total volume of 1 ml was used to test the hydrolytic activity in crude enzymes produced by *T. reesei*, which included 100 μl of 10 mg/ml 2′-FL and 900 μl of culture supernatants. Samples were boiled for 10 min to inactivate the enzymes and centrifuged at 12,000 *g* for 10 min. The supernatants were analyzed by HPLC equipped with an Aminex HPX-87H column (Bio-Rad, USA) and a RID-20A refractive index detector (Shimadzu, Japan). Elution was performed at 60°C using 5 mM H_2_SO_4_ as the mobile phase at a flow rate of 0.5 ml/min.

The β-d-glucuronidase, α-d-galactosidase, β-d-xylosidase, α-d-mannosidase, and α-l-fucosidase activities of proteins were measured using *p*-nitrophenyl-β-d-glucuronide (*p*NPβGlcA, Aladdin, China), *p*-nitrophenyl-α-d-galactopyranosideas (*p*NPαGal, Aladdin, China), *p*-nitrophenyl-β-d-xylopyranoside (*p*NPβX, Sigma-Aldrich, USA), *p*-nitrophenyl-α-d-mannopyranoside (*p*NPαM, Aladdin, China), and *p*-nitrophenyl-α-l-fucopyranoside (*p*NPαF, Aladdin, China) as the substrate, respectively. The reaction condition and product detection method are the same as those for β-d-xylosidase assay, as described previously [52]. One unit of enzyme activity was defined as the amount of enzyme that liberates 1 μmol *p*-nitrophenol from the substrate per minute.

### RNA-seq and RT-qPCR

Mycelia were harvested by vacuum filtration, ground in liquid nitrogen, and then used for extraction of total RNA using RNAiso Plus (TaKaRa, Japan). Further sample processing and high-throughput sequencing were performed by Personalbio Technology Co., Ltd. (Shanghai, China). The sequencing reads were mapped to the genome of *T. reesei* QM6a (NCBI assembly accession number: GCF_000167675.1), and FPKM (fragments per kilobase per million mapped fragments) was used to standardize the gene expression values. DESeq 1.38.3 [28] was used to identify the genes of significantly differential expression using the thresholds |log2FoldChange| ≥ 1 and FDR-adjusted *P*-value ≤0.001). For RT-qPCR, RNA was reverse-transcribed into the complementary DNA using HiScript III RT SuperMix for qPCR (+ gDNA wiper) (Vazyme, China). Then, qPCR analysis was conducted on LightCycler 480 II system (Roche, Switzerland) using TB Green *Premix Ex Taq* II (Tli RNaseH Plus) (TaKaRa, Japan) according to the manufacturer’s instructions. All PCR products had melting curves indicating the presence of a single amplicon. The transcript abundances were analyzed using the 2^−ΔΔ*C*^_*T*_ method using *sar1* gene as a reference [53,54]. The primers used for qPCR are listed in S5 Table.

### Electrophoretic mobility shift assay (EMSA)

EMSA was performed using Lightshift Chemiluminescent EMSA Kit (Thermo Scientific, USA) according to the manufacturer’s instructions. Briefly, 0.8 μg purified GST-FUR1BD and 20 fmol biotin-labeled dsDNA probe were incubated in binding buffer at 15°C for 25 min. Purified GST protein and 100-fold molar excess of unlabeled competitor DNA were added as indicated. The reaction mixtures were resolved by electrophoresis on 6% nondenaturing polyacrylamide gel at 95 V at 4°C, and then transferred to a nylon membrane at 300 mA for 30 min. After cross-linking, blocking, and incubation with streptavidin-horseradish peroxidase conjugate (1:300 dilution), the membrane was incubated with chemiluminescent substrate solution for 5 min, and analyzed by Tanon 4600 Chemiluminescent Imaging system (Tanon, China) for 1 min. the sequences of wide-type and mutated probes are 5’-CCCCATTATACCCGTCGGCTATTTACCAG-3’ and 5’-CCCCATTATACGGCTAAACTATTTACCAG-3’, respectively.

### Secretome analysis

The extracellular proteins of QMP and *fur1*-M were analyzed using label-free quantitative LC-MS/MS technique by Beijing Biomarker Technologies Co., Ltd. Briefly, proteins were digested with trypsin at 37°C for 2 h after denaturation and reductive alkylation. The peptides were desalted using a C18 column, concentrated by vacuum centrifugation, and reconstituted in 0.1% (v/v) formic acid. Then, LC-MS/MS analysis was performed on an Orbitrap Astral mass spectrometer coupled to Vanquish Neo UHPLC system (Thermo Scientific). The mass spectrometer was operated in positive mode, and MS data were acquired using a data-independent acquisition method. The MS raw data were searched against the *T. reesei* QM6a protein sequence database (NCBI BioProject: PRJNA225530), using DIA-NN 1.9 for protein identification and quantification [55]. Two maximum missed cleavages were allowed during the analysis, and FDR ≤ 0.01 was used for the screening at both peptide and protein levels. Proteins detected in all three replicate samples of one group but not detected in any of the replicate samples of the other group were defined as strain-unique proteins. The thresholds |log2FoldChange| ≥ 1 and *P*-value ≤ 0.05 were used to identify proteins with significantly different abundances between the two strains.

### Saccharification of orange peel

Orange peels were obtained from a local market in Qingdao, China, and cut into small pieces (1 × 1 cm). The peels were added with deionized water (solid/water ratio of 1:10) and then boiled for 20 min with slow stirring to remove the soluble pigments and dust. It was then dried at 60°C, ground into powder and passed through a 40-mesh sieve. The saccharification experiments were carried out in 50 ml Erlenmeyer flasks with a total reaction volume of 20 ml. The system consisted of orange peel powder at a concentration of 2% (w/v, dry weight), enzymes, and 0.05 M citric acid-sodium citrate buffer (pH 4.8). The mixture was incubated at 50°C with shaking at 150 rpm in a rotary shaker. The concentrations of glucose, galactose, xylose and arabinose in the supernatants were determined by HPLC as previously described [56].

### Bioinformatics analyses

Protein sequences of FUR1 homologues were retrieved from the NCBI database, aligned by Clustal Omega [57], and maximum likelihood tree was constructed using Molecular Evolutionary Genetics Analysis version 11 [58]. The NCBI RefSeq or GenPept accession numbers of the proteins are listed in S6 Table. Protein structures were predicted using the AlphaFold3 server [59], and visualized in PyMOL version 3.1.3 (Schrödinger, LLC). The 1200-bp sequences upstream of the start codon of FUR1 target genes were extracted from the genomic sequence by BEDTools version 2.31.1 [60], and used for motif discovery by MEME version 5.5.7 (motif site distribution: any number of sites per sequence; minimum motif width: 5; maximum motif width: 20) [61].

### Statistical analysis

The statistical significance values between samples were calculating using one-tailed homoscedastic *t*-test in the software Microsoft Office 2016 Excel. All the data are expressed as means ± SD.

## Supporting information

S1 Fig The effect of *fur1* deletion on the growth of *T. reesei* on various carbon sources. Strains were cultured on PDA or minimal medium with 0.5% (w/v) sugar as the carbon source for 5 days. No C, carbon source-free medium.
(DOCX)

S2 Fig Total ion chromatogram of the reaction products obtained from incubating l-fucose with FDH1 and NAD^+^. The same reaction using inactivated FDH1 (boiled for 5 min) was performed as a control.
(DOCX)

S3 Fig Characterization of purified FDH1. (A-C) Effect of pH, temperature and metal ions (1 mM) on the enzyme activity, respectively. (D) Determination of kinetic parameters of FDH1 with l-fucose as substrate. Data represent mean ± SD from triplicate reactions.
(DOCX)

S4 Fig Alignment of the sequences of l-fucose dehydrogenases from *Trichoderma reesei* (TrFDH1), *Burkholderia multivorans* (BMULJ_04919, UniProt accession: A0A0H3KNE7), and *Homo sapiens* (HSD17B14, UniProt accession: Q9BPX1). The residues interacting with NAD(P)^+^ are indicated by yellow background. The catalytic residues are indicated by red background. The residues interacting with l-fucose in the structure of BMULJ_04919 are indicated by black arrows.
(DOCX)

S5 Fig Relative activities of extracellular β-glucuronidase, α-galactosidase, β-xylosidase and α-mannosidase of QMP (set as 100%) and mutant strains in 0.5% (w/v) d-xylose medium after cultivation of 60 h. Data represent mean ± SD from triplicate cultivations.
(DOCX)

S6 Fig Alignment of the sequences of FUR1 and XYR1. The start and end residues of XYR1 fragments used for the construction of chimeric transcription factors are labeled.
(DOCX)

S7 Fig Schematic diagram of CRISPR/Cas9-aided construction of strains expressing chimeric transcription factors. Grey rectangles indicate regions for homologous recombination.
(DOCX)

S8 Fig The effect of *fur1* gene editing on the growth of *T. reesei.* Strains were cultured on minimal medium containing 0.5% (w/v) carbon source for 5 days.
(DOCX)

S9 Fig Relative activities of extracellular β-glucuronidase, α-galactosidase, β-xylosidase and α-mannosidase of QMP (set as 100%) and *fur1*-M. Strains were cultured in minimal medium with 0.5% (w/v) l-fucose as the carbon source for 60 h. ***, *P* < 0.001; **, *P* < 0.01; *, *P* < 0.05. Data represent mean ± SD from triplicate cultivations.
(DOCX)

S1 Table Comparison of the secretomes of QMP and *fur1*-M. (XLSX)

S2 Table Primers for the construction of *fur1* engineered strains. (DOCX)

S3 Table Primers for gene editing of *fur1* to express chimeric transcription factors. (DOCX)

S4 Table Primers for heterologous protein expression. (DOCX)

S5 Table Primers for qPCR. (DOCX)

S6 Table RefSeq or GenPept accession numbers of FUR1 homologues analyzed in this study. (DOCX)

S1 Data Re-annotation of genes *fur1*, *fdh1* and *afc95A.* (DOCX)

S2 Data Genes affected by *fur1* manipulations. (XLSX)
