# Supplementary material for: An l-fucose-responsive transcription factor cross-regulates the expression of a diverse array of carbohydrate-active enzymes in Trichoderma reesei
Source: PLoS Genet. 2025 Aug 11;21(8):e1011815. doi: 10.1371/journal.pgen.1011815 (PMC12370193; doi:10.1371/journal.pgen.1011815)
Supplement: S1 Fig — Strains were cultured on PDA or minimal medium with 0.5% (w/v) sugar as the carbon source for 5 days. No C, carbon source-free medium. (DOCX) [file pgen.1011815.s001.docx]

**
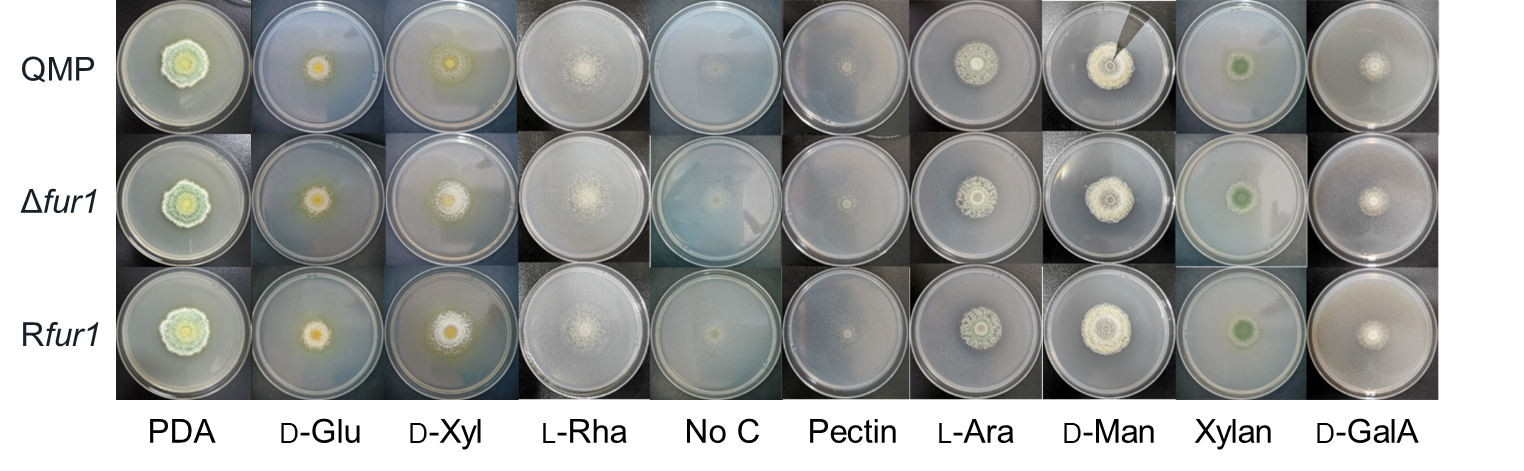
**

**S1 Fig. The effect of *fur1* deletion on the growth of *T. reesei* on various carbon sources.**

Strains were cultured on PDA or minimal medium with 0.5% (w/v) sugar as the carbon source for 5 days. No C, carbon source-free medium.
