# Supplementary material for: An l-fucose-responsive transcription factor cross-regulates the expression of a diverse array of carbohydrate-active enzymes in Trichoderma reesei
Source: PLoS Genet. 2025 Aug 11;21(8):e1011815. doi: 10.1371/journal.pgen.1011815 (PMC12370193; doi:10.1371/journal.pgen.1011815)
Supplement: S2 Fig — The same reaction using inactivated FDH1 (boiled for 5 min) was performed as a control. (DOCX) [file pgen.1011815.s002.docx]

**
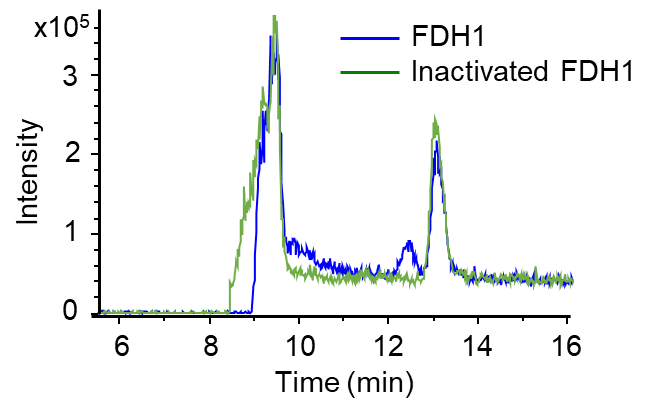
**

**S2 Fig. Total ion chromatogram of the reaction products obtained from incubating l-fucose with FDH1 and NAD^+^.**

The same reaction using inactivated FDH1 (boiled for 5 min) was performed as a control.
