# Supplementary material for: An l-fucose-responsive transcription factor cross-regulates the expression of a diverse array of carbohydrate-active enzymes in Trichoderma reesei
Source: PLoS Genet. 2025 Aug 11;21(8):e1011815. doi: 10.1371/journal.pgen.1011815 (PMC12370193; doi:10.1371/journal.pgen.1011815)
Supplement: S3 Fig — (A-C) Effect of pH, temperature and metal ions (1 mM) on the enzyme activity, respectively. (D) Determination of kinetic parameters of FDH1 with l-fucose as substrate. Data represent mean ± SD from triplicate reactions. (DOCX) [file pgen.1011815.s003.docx]

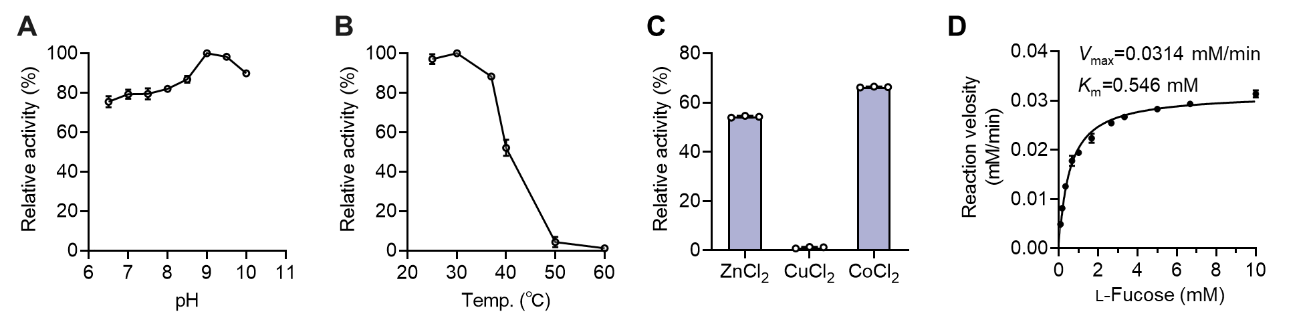


**S3 Fig. Characterization of purified FDH1.**

(**A-C**) Effect of pH, temperature and metal ions (1 mM) on the enzyme activity, respectively. (**D**) Determination of kinetic parameters of FDH1 with l-fucose as substrate. Data represent mean ± SD from triplicate reactions.
