# Supplementary material for: An l-fucose-responsive transcription factor cross-regulates the expression of a diverse array of carbohydrate-active enzymes in Trichoderma reesei
Source: PLoS Genet. 2025 Aug 11;21(8):e1011815. doi: 10.1371/journal.pgen.1011815 (PMC12370193; doi:10.1371/journal.pgen.1011815)
Supplement: S4 Fig — The residues interacting with NAD(P)+ are indicated by yellow background. The catalytic residues are indicated by red background. The residues interacting with l-fucose in the structure of BMULJ_04919 are indicated by black arrows. (DOCX) [file pgen.1011815.s004.docx]

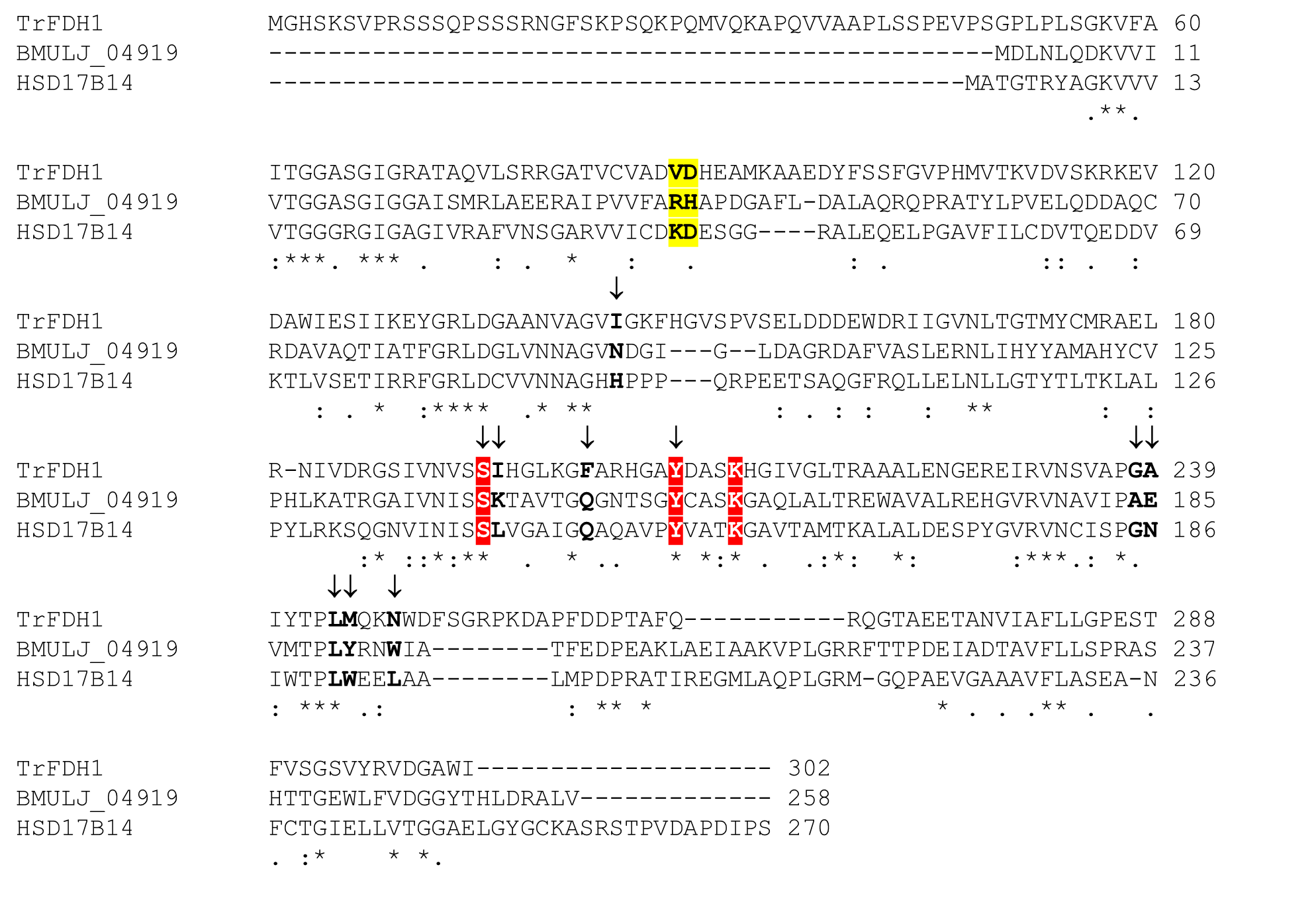


**S4 Fig. Alignment of the sequences of l-fucose dehydrogenases from *Trichoderma reesei* (TrFDH1), *Burkholderia multivorans* (BMULJ_04919, UniProt accession: A0A0H3KNE7), and *Homo sapiens* (HSD17B14, UniProt accession: Q9BPX1).**

The residues interacting with NAD(P)^+^ are indicated by yellow background. The catalytic residues are indicated by red background. The residues interacting with l-fucose in the structure of BMULJ_04919 are indicated by black arrows.
