# Supplementary material for: An l-fucose-responsive transcription factor cross-regulates the expression of a diverse array of carbohydrate-active enzymes in Trichoderma reesei
Source: PLoS Genet. 2025 Aug 11;21(8):e1011815. doi: 10.1371/journal.pgen.1011815 (PMC12370193; doi:10.1371/journal.pgen.1011815)
Supplement: S5 Fig — Data represent mean ± SD from triplicate cultivations. (DOCX) [file pgen.1011815.s005.docx]

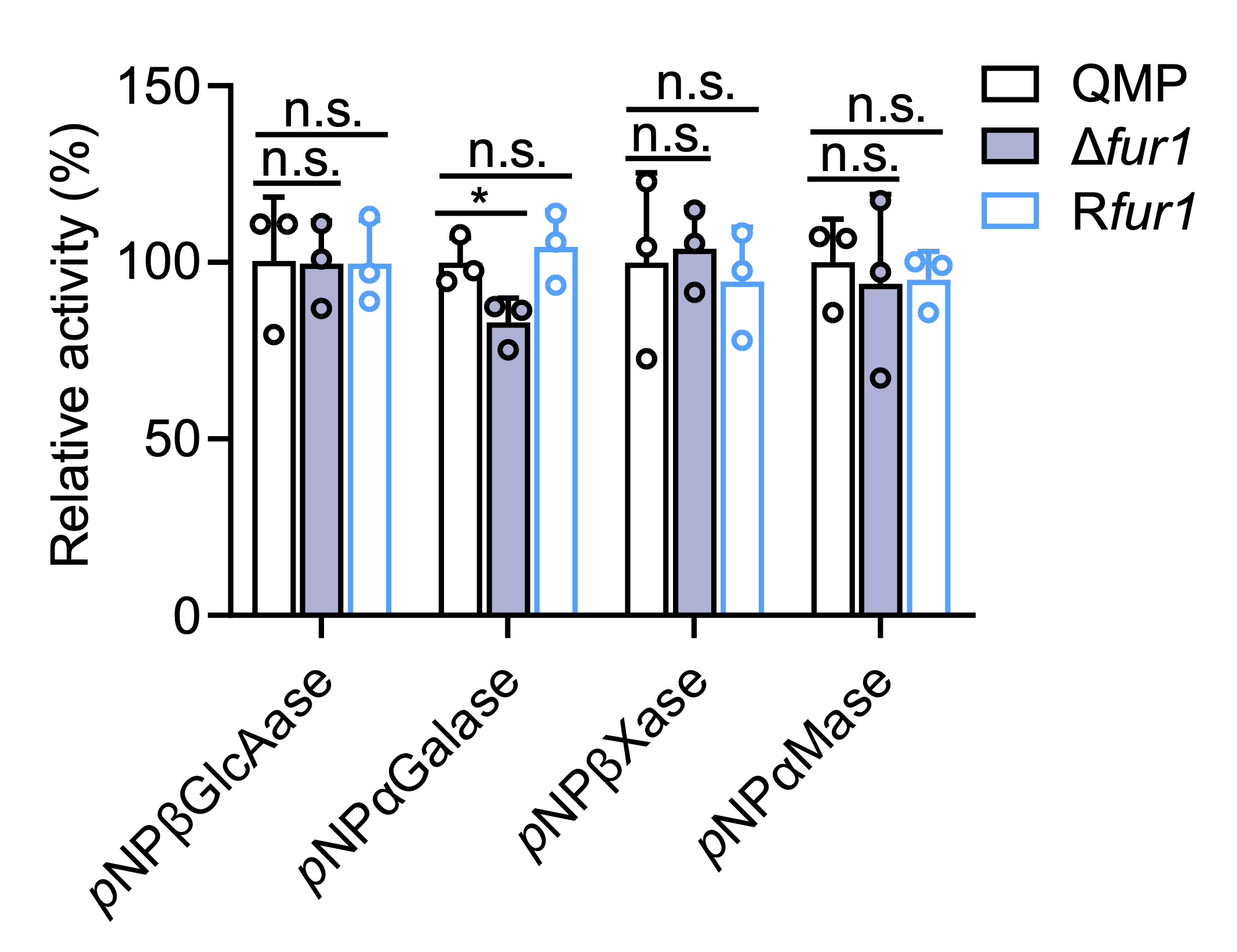


**S5 Fig. Relative activities of extracellular β-glucuronidase, α-galactosidase, β-xylosidase and α-mannosidase of QMP (set as 100%) and mutant strains in 0.5% (w/v) d-xylose medium after cultivation of 60 h.**

Data represent mean ± SD from triplicate cultivations.
