# Supplementary material for: An l-fucose-responsive transcription factor cross-regulates the expression of a diverse array of carbohydrate-active enzymes in Trichoderma reesei
Source: PLoS Genet. 2025 Aug 11;21(8):e1011815. doi: 10.1371/journal.pgen.1011815 (PMC12370193; doi:10.1371/journal.pgen.1011815)
Supplement: S6 Fig — The start and end residues of XYR1 fragments used for the construction of chimeric transcription factors are labeled. (DOCX) [file pgen.1011815.s006.docx]

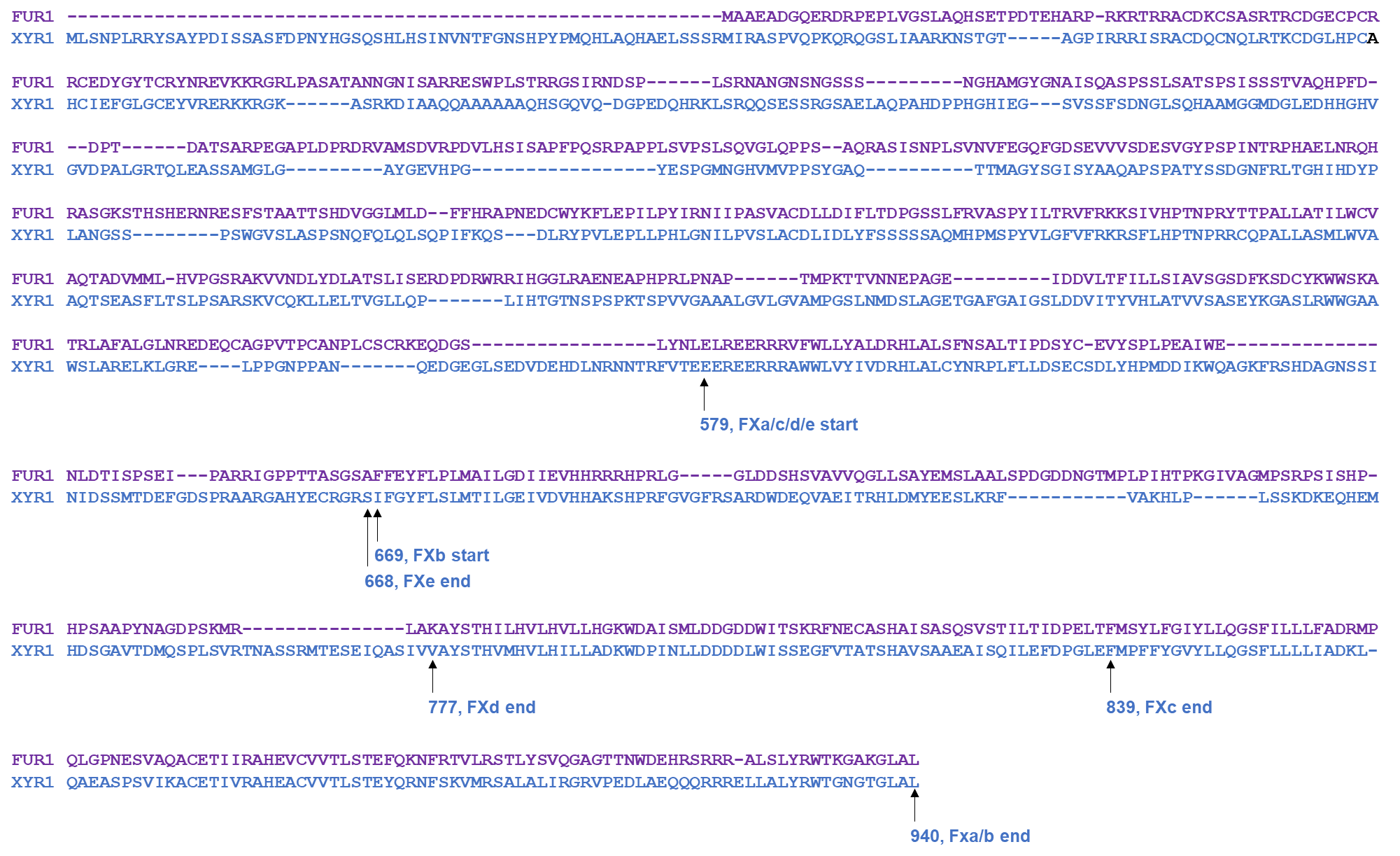


**S6 Fig. Alignment of the sequences of FUR1 and XYR1.**

The start and end residues of XYR1 fragments used for the construction of chimeric transcription factors are labeled.
