# Supplementary material for: An l-fucose-responsive transcription factor cross-regulates the expression of a diverse array of carbohydrate-active enzymes in Trichoderma reesei
Source: PLoS Genet. 2025 Aug 11;21(8):e1011815. doi: 10.1371/journal.pgen.1011815 (PMC12370193; doi:10.1371/journal.pgen.1011815)
Supplement: S7 Fig — Grey rectangles indicate regions for homologous recombination. (DOCX) [file pgen.1011815.s007.docx]

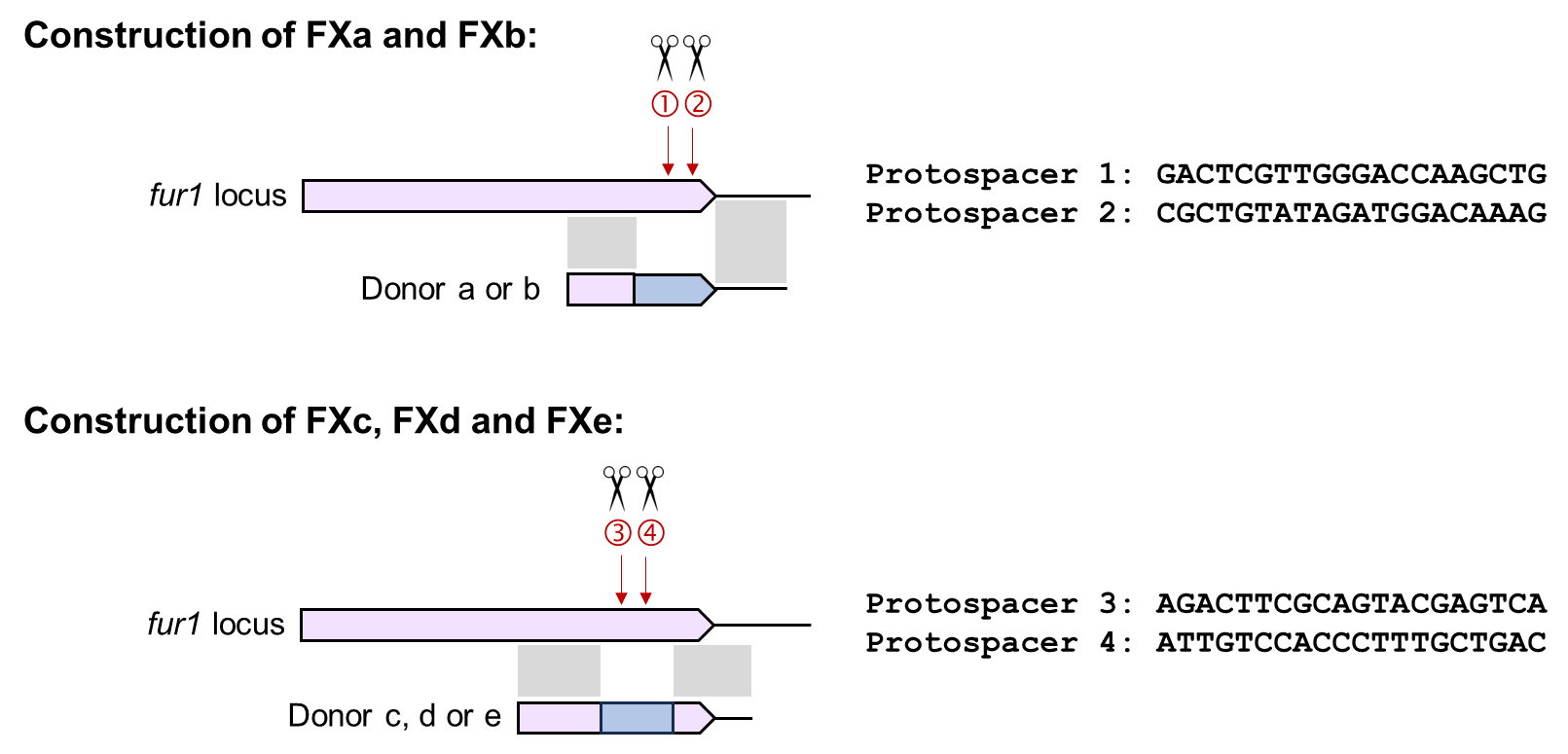


**S7 Fig. Schematic diagram of CRISPR/Cas9-aided construction of strains expressing chimeric transcription factors.**

Grey rectangles indicate regions for homologous recombination.
