# Supplementary material for: An l-fucose-responsive transcription factor cross-regulates the expression of a diverse array of carbohydrate-active enzymes in Trichoderma reesei
Source: PLoS Genet. 2025 Aug 11;21(8):e1011815. doi: 10.1371/journal.pgen.1011815 (PMC12370193; doi:10.1371/journal.pgen.1011815)
Supplement: S8 Fig — Strains were cultured on minimal medium containing 0.5% (w/v) carbon source for 5 days. (DOCX) [file pgen.1011815.s008.docx]

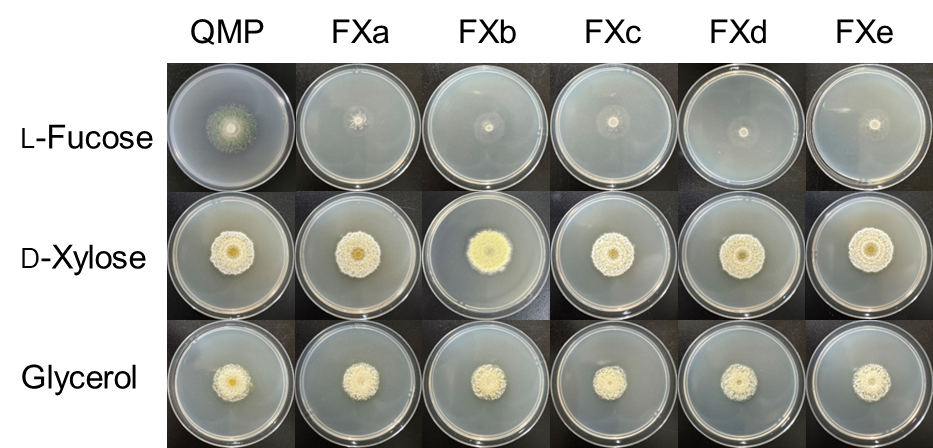


**S8 Fig. The effect of *fur1* gene editing on the growth of *T. reesei*.**

Strains were cultured on minimal medium containing 0.5% (w/v) carbon source for 5 days.
