# Supplementary material for: An l-fucose-responsive transcription factor cross-regulates the expression of a diverse array of carbohydrate-active enzymes in Trichoderma reesei
Source: PLoS Genet. 2025 Aug 11;21(8):e1011815. doi: 10.1371/journal.pgen.1011815 (PMC12370193; doi:10.1371/journal.pgen.1011815)
Supplement: S9 Fig — Strains were cultured in minimal medium with 0.5% (w/v) l-fucose as the carbon source for 60 h. ***, P < 0.001; **, P < 0.01; *, P < 0.05. Data represent mean ± SD from triplicate cultivations. (DOCX) [file pgen.1011815.s009.docx]

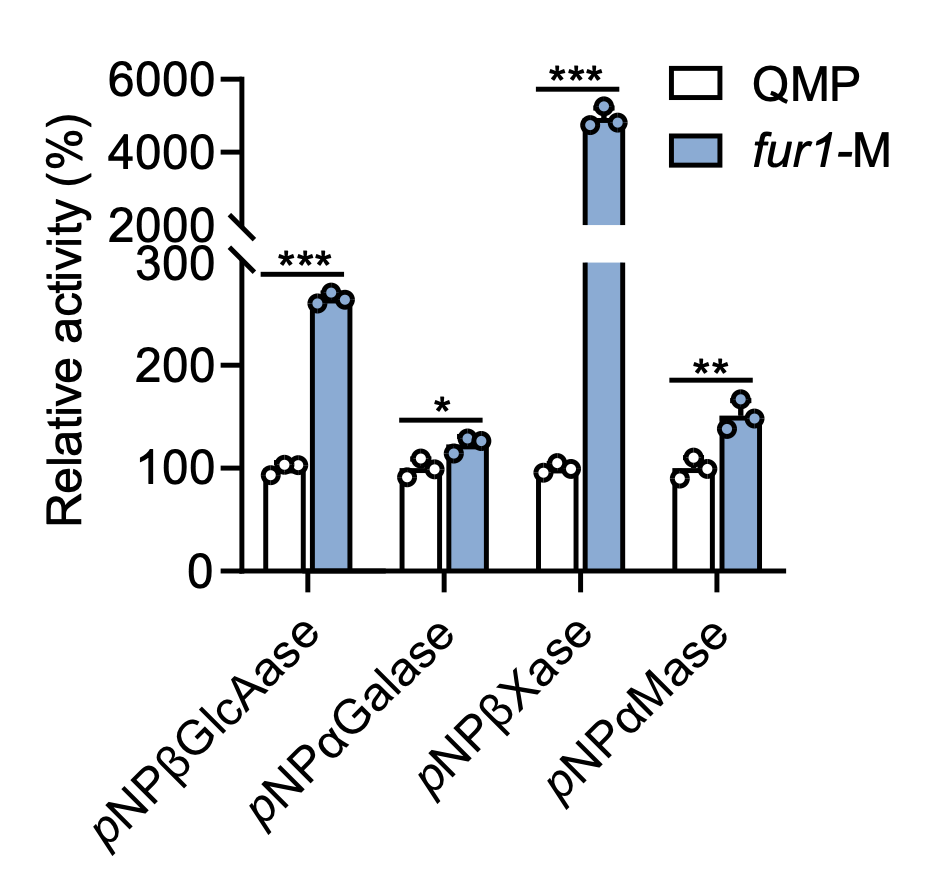


**S9 Fig. Relative activities of extracellular β-glucuronidase, α-galactosidase, β-xylosidase and α-mannosidase of QMP (set as 100%) and *fur1*-M.**

Strains were cultured in minimal medium with 0.5% (w/v) l-fucose as the carbon source for 60 h. ***, *P* < 0.001; **, *P* < 0.01; *, *P* < 0.05. Data represent mean ± SD from triplicate cultivations.
