# Supplementary material for: An l-fucose-responsive transcription factor cross-regulates the expression of a diverse array of carbohydrate-active enzymes in Trichoderma reesei
Source: PLoS Genet. 2025 Aug 11;21(8):e1011815. doi: 10.1371/journal.pgen.1011815 (PMC12370193; doi:10.1371/journal.pgen.1011815)
Supplement: S2 Table — (DOCX) [file pgen.1011815.s011.docx]

**S2 Table.** Primers for the construction of *fur1* engineered strains.

| **Primers** | **Sequence (5′–3′)** | **Product** |
| --- | --- | --- |
| **Construction of Δ*fur1*:** | | |
| FUR1-UF | GTGAGTGGGAGCGTGTATCGCGTG | The left arm of *fur1* deletion cassette (together with FUR1-UR) |
| FUR1-UR | CTGCGGCGCGTTCTCGAGGAAGTTGCGATCCTTCTCGCGTGTTATCTCTGCC |  |
| FUR1-DF | GCTTACGAGAAAAGAGTTGGACTTTGAGGATGGGTTATGGATGGCATCAAGTC | The right arm of *fur1* deletion cassette (together with FUR1-DR) |
| FUR1-DR | GATGAGGCGCTCGACTTCAAAATGC |  |
| pyrG-F | CAACTTCCTCGAGAACGCGCCGCAG | The selection marker gene *pyrG* (together with pyrG-R) |
| pyrG-R | TCAAAGTCCAACTCTTTTCTC |  |
| **Construction of R*fur1*:** | | |
| Pfur1-F | GTGAGTGGGAGCGTGTATCGCG | The promoter, coding region and terminator of FUR1 (together with Tfur1-R) |
| Tfur1-R | GCTCCTTCAATATCAGTTAACCCAAGCAAAGGCTCAACCACACTCTCC |  |
| hph-F | GTTAACTGATATTGAAGGAGC | The selection marker gene *hph* (together with hph-R) |
| hph-R | CAACCCAGGGCTGGTGACGG |  |
| **Construction of *fur1*-M:** | | |
| Pcdna1-F | TGGTACATGGATCTCGAACTG | The promoter of *cdna1* (together with Pcdna1-R) |
| Pcdna1-R | GTTGAGAGAAGTTGTTGGATTG |  |
| FUR1-F | CAATCCAACAACTTCTCTCAACATGGCAGCTGAAGCCGACGGCCAAG | Upstream sequence of mutated sites of *fur1*, together with FUR1-MR |
| FUR1-MR | GACCGCAGAGAAGGCATGCGACGCGCATTCGTTGAAC |  |
| FUR1-MF | GGTTCAACGAATGCGCGTCGCATGCCTTCTCTGCGGTCCAGTCCGTCTCGACTATC | Downstream sequence of mutated sites of *fur1*, together with FUR1-R |
| FUR1-R | GATGATTTCAGTAACGTTAAGTGGATCTCACAATGCCAGTCCCTTGGCCCCCTTTG |  |
| TrpC-F | GATCCACTTAACGTTACTGAAATCATC | The terminator sequence of *A. nidulans* *trpC* and selection marker gene *pyrG* (together with pyrG-R). |
| pyrG-R | TCAAAGTCCAACTCTTTTCTC |  |
