# Supplementary material for: An l-fucose-responsive transcription factor cross-regulates the expression of a diverse array of carbohydrate-active enzymes in Trichoderma reesei
Source: PLoS Genet. 2025 Aug 11;21(8):e1011815. doi: 10.1371/journal.pgen.1011815 (PMC12370193; doi:10.1371/journal.pgen.1011815)
Supplement: S3 Table — (DOCX) [file pgen.1011815.s012.docx]

**S3 Table.** Primers for gene editing of *fur1* to express chimeric transcription factors.

| **Primers** | **Sequence (5′–3′)** | **Product** |
| --- | --- | --- |
| Fx-FUR1-F | CAACTCGCCGTGGAAGCATCC | The left arm of donors a,c,d and e (together with Fx-FUR1-R) |
| Fx-FUR1-R | GAGATTGTACAAGCTACCATCCTGC |  |
| XYR1-F | TCGAAAAGAGCAGGATGGTAGCTTGTACAATCTCGAGGAGCGCGAAGAGCGACGGC | Partial *xyr1* sequence for donors a,c,d and e |
| XYR1-ab-R | CCATCGTGACTTGATGCCATCCATAACCCATCCTTAGAGGGCCAGACCGGTTCCGTT | Partial *xyr1* sequence for donor a (together with XYR1-F) |
| Tfur1-F | GGACTGGTAACGGAACCGGTCTGGCCCTCTAAGGATGGGTTATGGATGGCATCAAG | The right arm of donors a and b (together with Fx-Tfur1-R) |
| Fx-Tfur1-R | GAGCAATGTATGTGGTGATTACT |  |
| FUR1-b-R | CATCAAGGACAAGAAGTAGCCAAAAATTGCAGATCCAGAGGCAGTCGTG | The left arm of donor b (together with Fx-FUR1-F) |
| XYR1-b-F | GACTGCCTCTGGATCTGCAATTTTTGGCTACTTCTTGTCCTTG | Partial *xyr1* sequence for donor b (together with XYR1-ab-R) |
| XYR1-c-R | AAACTCCAGGCCAGGGTCAAAC | Partial *xyr1* sequence for donor c (together with XYR1-F) |
| FUR1-c-DF | TCTCGAGTTTGACCCTGGCCTGGAGTTTATGTCCTATCTCTTCGGGATCTATCTG | The right arm of donor c (together with Fx-Tfur1-R) |
| XYR1-d-R | CACCACGATGCTGGCCTGGATC | Partial *xyr1* sequence for donor d (together with XYR1-F) |
| FUR1-d-DF | CGAGATCCAGGCCAGCATCGTGGTGGCCTATAGCACGCACATCTTGCACGTG | The right arm of donor d (together with Fx-Tfur1-R) |
| XYR1-e-R | GCTACGACCGCGGCACTCGTAG | Partial *xyr1* sequence for donor e (together with XYR1-F) |
| FUR1-e-DF | CACACTACGAGTGCCGCGGTCGTAGCTTTTTCGAGTACTTTTTACCCCTCATG | The right arm of donor e (together with Fx-Tfur1-R) |
| sgRNA-ab-F1 | GACTCGTTGGGACCAAGCTGGTTTTAGAGCTAGAAATAGCAAG | sgRNA array for the construction of FXa and FXb strains (F1 and R1 for first-round PCR, and F2 and R2 for second-round PCR) |
| sgRNA-ab-R1 | CTTTGTCCATCTATACAGCGTGCGTAATCTGGGAATCGAACCC |  |
| sgRNA-ab-F2 | AGCCCTGGGTTCGATTCCCAGATTACGCAGACTCGTTGGGACCAAGCTGGTT |  |
| sgRNA-ab-R2 | CTTATTTTAACTTGCTATTTCTAGCTCTAAAACCTTTGTCCATCTATACAGCGTG |  |
| sgRNA-cde-F1 | GCAAGACTTCGCAGTACGAGTCAGTTTTAGAGCTAGAAATAGCAAG | sgRNA array for the construction of FXc, FXd and FXe strains (F1 and R1 for first-round PCR, and F2 and R2 for second-round PCR) |
| sgRNA-cde-R1 | AACGTCAGCAAAGGGTGGACAATTGCGTAATCTGGGAATCGAACCC |  |
| sgRNA-cde-F2 | GAGCCCTGGGTTCGATTCCCAGATTACGCAAGACTTCGCAGTACGAGTCAG |  |
| sgRNA-cde-R2 | CTTATTTTAACTTGCTATTTCTAGCTCTAAAACGTCAGCAAAGGGTGGACAAT |  |
