# Supplementary material for: An l-fucose-responsive transcription factor cross-regulates the expression of a diverse array of carbohydrate-active enzymes in Trichoderma reesei
Source: PLoS Genet. 2025 Aug 11;21(8):e1011815. doi: 10.1371/journal.pgen.1011815 (PMC12370193; doi:10.1371/journal.pgen.1011815)
Supplement: S4 Table — (DOCX) [file pgen.1011815.s013.docx]

**S4 Table.** Primers for heterologous protein expression.

| **Primers** | **Sequence (5′–3′)** | **Product** |
| --- | --- | --- |
| FUR1-4T-F | GATCTGGTTCCGCGTGGATCCCCGGAATTCGCCCGGCCGAGGAAGCGGACCCGGAGG | The coding region of DNA binding domain of FUR1 |
| FUR1-4T-R | CAGATCGTCAGTCAGTCACGATGCGGCCGCCTATCGGGCGCTGATGTTGCCGTTG |  |
| FDH1-32a-F | GACGACAAGGCCATGGCTGATATCGGATCCGAATTCATGGGCCATAGCAAAAGCGTGC | The coding sequence of FDH1 (codon optimized) |
| FDH1-32a-R | CAGTGGTGGTGGTGGTGGTGCTCGAGTGCGGCCGCTTAAATCCACGCGCCATCCACGC |  |
| Tr5807-F | GAGAGGCTGAAGCTTACGTAAGGAAACTATGGGCAACTGAACC | The coding region of Afc95A mature protein |
| Tr5807-R | TCTAAGGCGAATTAATTCGCTCAATGGTGATGGTGATGATGCTGATGAATCAAGACCTTGCCG |  |
| Tr58802-F | GAGAGGCTGAAGCTTACGTAGCCCTGGACGGAAGCCGATATC | The coding region of Afc95B mature protein |
| Tr58802-R | TCTAAGGCGAATTAATTCGCTCAATGGTGATGGTGATGATGAACAGTAATGGAATACTTGGTTC |  |
