# Supplementary material for: An l-fucose-responsive transcription factor cross-regulates the expression of a diverse array of carbohydrate-active enzymes in Trichoderma reesei
Source: PLoS Genet. 2025 Aug 11;21(8):e1011815. doi: 10.1371/journal.pgen.1011815 (PMC12370193; doi:10.1371/journal.pgen.1011815)
Supplement: S5 Table — (DOCX) [file pgen.1011815.s014.docx]

**S5 Table.** Primers for qPCR.

| **Primers** | **Sequence (5′–3′)** |
| --- | --- |
| fdh1-qPCR-F | AAGGCTGCCGAGGACTATTT |
| fdh1-qPCR-R | AAACTTTCCAATGACTCCTGCTA |
| lgd1-qPCR-F | TACACCCAGCATCTGAGCACC |
| lgd1-qPCR-R | CCACCAACTTACGCCCTTCTT |
| lga1-qPCR-F | TTGGCGGCATGTGCGACTT |
| lga1-qPCR-R | CGCCGAGGATAGCCTCCGTAT |
| gar1-qPCR-F | GAACATGGCAGTCCAAAGC |
| gar1-qPCR-R | TATCCAGGCCCAGCACAAC |
| 73005-qPCR-F | ATGCCACTGTCGAGCTGAT |
| 73005-qPCR-R | TGTGAATGCCGCACCTGAA |
| afc95a-qPCR-F | CTTCTCCGTCTACACCACAGG |
| afc95a-qPCR-R | ACCAAGCCATTATCGTCCC |
| afc95b-qPCR-F | GTCCACCTGCTGCCTGCTCT |
| afc95b-qPCR-R | GGTCGAGATTGGTCCATTGTATT |
| 72704-qPCR-F | CTTCCGCAAAGATGGTGTC |
| 72704-qPCR-R | CAAGGTCGCTCCAGATAGGT |
| 58450-qPCR-F | CAGACTACCCGTGACTCAATAC |
| 58450-qPCR-R | ACTGGAACGACAGGGAGAA |
| sar1-qPCR-R | TGGTGTATGTTATTGGGATT |
| sar1-qPCR-R | ACACCACGAGAAGTCAAGA |
