# Supplementary material for: An l-fucose-responsive transcription factor cross-regulates the expression of a diverse array of carbohydrate-active enzymes in Trichoderma reesei
Source: PLoS Genet. 2025 Aug 11;21(8):e1011815. doi: 10.1371/journal.pgen.1011815 (PMC12370193; doi:10.1371/journal.pgen.1011815)
Supplement: S6 Table — (DOCX) [file pgen.1011815.s015.docx]

**S6 Table.** RefSeq or GenPept accession numbers of FUR1 homologues analyzed in this study.

| **Species** | **Protein ID** | **Accession No.** | **Note** |
| --- | --- | --- | --- |
| *Trichoderma reesei* | TRIREDRAFT_122208 | XP_006966092.1 | Corrected by adding VSLASPSNQFQLQLSQPIFK after G319 |
| *Trichoderma reesei* | TRIREDRAFT_60282 | XP_006964572.1 | Corrected in this study |
| *Aspergillus niger* | An15g05810 | XP_001397110.3 |  |
| *Aspergillus niger* | An11g06290 | XP_001394612.3 |  |
| *Aspergillus niger* | An04g08600 | XP_059600664.1 |  |
| *Fusarium oxysporum* | FOBCDRAFT_220915 | XP_059464672.1 |  |
| *Fusarium oxysporum* | FOBCDRAFT_157991 | XP_031048931.2 |  |
| *Metarhizium robertsii* | MAA_01135 | XP_007817324.2 |  |
| *Metarhizium robertsii* | MAA_11670 | XP_011410887.1 |  |
| *Penicillium oxalicum* | PDE_04461 | EPS29511.1 |  |
| *Penicillium oxalicum* | PDE_07674 | EPS32714.1 |  |
| *Penicillium oxalicum* | PDE_07172 | EPS32212.1 |  |
