# Supplementary material for: An l-fucose-responsive transcription factor cross-regulates the expression of a diverse array of carbohydrate-active enzymes in Trichoderma reesei
Source: PLoS Genet. 2025 Aug 11;21(8):e1011815. doi: 10.1371/journal.pgen.1011815 (PMC12370193; doi:10.1371/journal.pgen.1011815)
Supplement: S1 Data — (DOCX) [file pgen.1011815.s016.docx]

**S1 Data.** Re-annotation of genes *fur1*, *fdh1* and *afc95A*.

**1. FUR1 (TRIREDRAFT_60282)**

**Gene sequence, introns in lower case:**

ATGGCAGCTGAAGCCGACGGCCAAGAGCGGGATCGCCCCGAGCCTCTTGTCGGCTCATTGGCGCAGCATAGCGAGACGCCCGATACGGAGCACGCCCGGCCGAGGAAGCGGACCCGGAGGGCTTGCGACAAGTGCAGCGCCTCAAGGACGCGGTGCGATGGCGAgtggtgagttgggacggttcatggtttctgcatatcagtgatggtgtctaaccgggacatttgtgcgcgtcagTCCTTGTCGTCGGTGCGAAGgtaagcaccatgtctcttccatttgtccttcttcctggaatgcggcaacccccctcttctcattgtctcacctcataccaatcatacatgtaccttctctaacatgctgcctctgccatcaagACTACGGATACACCTGTCGTTATAACCGTGAAGTCAAGAAGAGAGGCCGTCTACCGGCCTCGGCTACGGCCAACAACGGCAACATCAGCGCCCGAAGGGAATCCTGGCCGTTGTCAACTCGCCGTGGAAGCATCCGCAACGACTCTCCCCTGAGTCGCAACGCCAATGGCAATAGCAACGGCAGCAGCAGCAACGGCCACGCCATGGGCTACGGCAATGCCATCTCGCAGGCCTCGCCCTCGTCTCTGTCAGCCACCTCGCCCAGCATCAGCTCTAGCACGGTCGCACAGCATCCCTTTGATGACCCAACGGATGCAACCTCGGCTCGTCCCGAAGGTGCACCCCTGGATCCGAGGGATCGGGTCGCCATGTCCGACGTTCGCCCAGACGTGCTGCATTCCATCAGCGCACCCTTTCCCCAGAGTCGACCCGCCCCTCCGCTCAGCGTCCCGTCTCTCAGCCAAGTTGGACTCCAACCGCCTTCTGCGCAGCGCGCTTCCATAAGCAACCCCCTGTCTGTGAATGTGTTTGAGGGGCAGTTTGGGGACTCGGAAGTCGTCGTGTCCGATGAGAGCGTTGGATACCCGTCACCGATAAACACCAGGCCCCATGCCGAGCTGAACCGCCAACACAGGGCGTCCGGCAAGTCGACGCACAGCCACGAGCGGAACAGAGAGTCATTCAGCACAGCGGCAACGACCAGCCACGATGTCGGGGGCCTGATGTTGGATTTCTTCCACAGAGCCCCAAACGAGGACTGCTGGTACAAGTTTCTGGAGCCCATCTTGCCGTACATCCGCAACATCATCCCCGCCTCTGTTGCGTGCGACTTGCTCGACATATTTCTGACGGATCCGGGAAGCTCGCTGTTTCGTGTTGCGTCCCCCTATATCCTGACTAGGGTCTTTCGTAAAAAGTCCATTGTGCACCCGACGAACCCCAGATACACCACCCCGGCGCTGCTGGCGACCATACTCTGGTGTGTAGCACAGACTGCCGATGTCATGATGCTGCATGTCCCGGGCTCCAGGGCCAAGGTTGTCAACGATCTCTACGACCTAGCAACGTCCCTGATATCCGAGCGTGACCCGGACCGGTGGCGACGAATTCATGgtaggccattgttaacggaaattgacgatgtgggacgcatattgacatatcccagGTGGTCTGCGTGCAGAAAATGAAGCACCTCACCCTCGGCTTCCTAATGCGCCGACAATGCCAAAGACAACAGTCAACAACGAGCCAGCGGGCGAGATCGATGATGTTCTCACCTTCATCCTCCTCTCCATTGCCGTCTCGGGCTCCGATTTCAAGTCGGACTGCTACAAGTGGTGGTCCAAGGCAACGCGACTTGCCTTTGCGCTGGGCCTCAACCGAGAGGACGAGCAATGCGCCGGACCCGTGACGCCCTGCGCGAATCCTCTCTGCTCGTGTCGAAAAGAGCAGGATGGTAGCTTGTACAATCTCGAGCTGCGAGAAGAGCGCCGGCGAGTCTTTTGGCTTCTCTATGCCTTGGACCGGCACTTGGCCCTGTCTTTCAACTCTGCCCTTACCATCCCTGACTCGTACTGCGAAGTCTACTgtaaggaccaaaacctgcccccaatctgttcattgtccaccctttgctgaccggcctcagCACCTCTTCCCGAAGCCATCTGGGAGAACCTCGACACGATTTCGCCCAGTGAGATTCCCGCCCGAAGAATCGGACCGCCCACGACTGCCTCTGGATCTGCATTTTTCGAGTACTTTTTACCCCTCATGGCGATACTAGGCGACATTATAGAGGTCCATCATCGGCGCCGTCACCCGAGACTCGGAGGCCTCGACGATTCACACTCCGTGGCCGTCGTTCAGGGGCTGCTTTCGGCCTACGAAATGAGTCTCGCCGCGCTGTCACCAGACGGCGACGACAACGGCACCATGCCCCTGCCTATTCACACGCCCAAAGGGATCGTTGCCGGAATGCCTTCCAGGCCATCCATTTCCCATCCACACCCGTCCGCAGCCCCTTACAACGCAGGCGATCCCTCCAAGATGCGGCTGGCAAAGGCCTATAGCACGCACATCTTGCACGTGCTGCACGTGCTGCTCCATGGAAAATGGGATGCCATCTCCATGCTGGACGACGGCGACGATTGGATCACGTCCAAAAGGTTCAACGAATGCGCGTCGCATGCCATTTCTGCGTCGCAGTCCGTCTCGACTATCTTGACTATCGATCCGGAACTGACCTTCATGTCCTATCTCTTCGGGATCTATCTGCTGCAGGGGAGTTTCATTCTTTTGCTCTTCGCGGACAGGATGCCGCAGCTTGGTCCCAACGAGTCGGTTGCGCAGGCGTGTGAGACCATTATTCGAGCTCACGAGGTGTGCGTTGTGACGCTGAGCACAGAATTCCAGgtatgcagccctcgtcccttttatgtgagaaatgctgcaggatgttgacatttagctagAAAAACTTTCGTACCGTTTTGCGATCTACGCTTTATAGTGTTCAAGGTGCAGGAACTACGAATTGGGACGAGCATCGTTCACGACGCAGAGCTTTGTCGCTGTATAGATGGACAAAGGGGGCCAAGGGACTGGCATTGTGA

**Coding sequence:**

ATGGCAGCTGAAGCCGACGGCCAAGAGCGGGATCGCCCCGAGCCTCTTGTCGGCTCATTGGCGCAGCATAGCGAGACGCCCGATACGGAGCACGCCCGGCCGAGGAAGCGGACCCGGAGGGCTTGCGACAAGTGCAGCGCCTCAAGGACGCGGTGCGATGGCGAGTGTCCTTGTCGTCGGTGCGAAGACTACGGATACACCTGTCGTTATAACCGTGAAGTCAAGAAGAGAGGCCGTCTACCGGCCTCGGCTACGGCCAACAACGGCAACATCAGCGCCCGAAGGGAATCCTGGCCGTTGTCAACTCGCCGTGGAAGCATCCGCAACGACTCTCCCCTGAGTCGCAACGCCAATGGCAATAGCAACGGCAGCAGCAGCAACGGCCACGCCATGGGCTACGGCAATGCCATCTCGCAGGCCTCGCCCTCGTCTCTGTCAGCCACCTCGCCCAGCATCAGCTCTAGCACGGTCGCACAGCATCCCTTTGATGACCCAACGGATGCAACCTCGGCTCGTCCCGAAGGTGCACCCCTGGATCCGAGGGATCGGGTCGCCATGTCCGACGTTCGCCCAGACGTGCTGCATTCCATCAGCGCACCCTTTCCCCAGAGTCGACCCGCCCCTCCGCTCAGCGTCCCGTCTCTCAGCCAAGTTGGACTCCAACCGCCTTCTGCGCAGCGCGCTTCCATAAGCAACCCCCTGTCTGTGAATGTGTTTGAGGGGCAGTTTGGGGACTCGGAAGTCGTCGTGTCCGATGAGAGCGTTGGATACCCGTCACCGATAAACACCAGGCCCCATGCCGAGCTGAACCGCCAACACAGGGCGTCCGGCAAGTCGACGCACAGCCACGAGCGGAACAGAGAGTCATTCAGCACAGCGGCAACGACCAGCCACGATGTCGGGGGCCTGATGTTGGATTTCTTCCACAGAGCCCCAAACGAGGACTGCTGGTACAAGTTTCTGGAGCCCATCTTGCCGTACATCCGCAACATCATCCCCGCCTCTGTTGCGTGCGACTTGCTCGACATATTTCTGACGGATCCGGGAAGCTCGCTGTTTCGTGTTGCGTCCCCCTATATCCTGACTAGGGTCTTTCGTAAAAAGTCCATTGTGCACCCGACGAACCCCAGATACACCACCCCGGCGCTGCTGGCGACCATACTCTGGTGTGTAGCACAGACTGCCGATGTCATGATGCTGCATGTCCCGGGCTCCAGGGCCAAGGTTGTCAACGATCTCTACGACCTAGCAACGTCCCTGATATCCGAGCGTGACCCGGACCGGTGGCGACGAATTCATGGTGGTCTGCGTGCAGAAAATGAAGCACCTCACCCTCGGCTTCCTAATGCGCCGACAATGCCAAAGACAACAGTCAACAACGAGCCAGCGGGCGAGATCGATGATGTTCTCACCTTCATCCTCCTCTCCATTGCCGTCTCGGGCTCCGATTTCAAGTCGGACTGCTACAAGTGGTGGTCCAAGGCAACGCGACTTGCCTTTGCGCTGGGCCTCAACCGAGAGGACGAGCAATGCGCCGGACCCGTGACGCCCTGCGCGAATCCTCTCTGCTCGTGTCGAAAAGAGCAGGATGGTAGCTTGTACAATCTCGAGCTGCGAGAAGAGCGCCGGCGAGTCTTTTGGCTTCTCTATGCCTTGGACCGGCACTTGGCCCTGTCTTTCAACTCTGCCCTTACCATCCCTGACTCGTACTGCGAAGTCTACTCACCTCTTCCCGAAGCCATCTGGGAGAACCTCGACACGATTTCGCCCAGTGAGATTCCCGCCCGAAGAATCGGACCGCCCACGACTGCCTCTGGATCTGCATTTTTCGAGTACTTTTTACCCCTCATGGCGATACTAGGCGACATTATAGAGGTCCATCATCGGCGCCGTCACCCGAGACTCGGAGGCCTCGACGATTCACACTCCGTGGCCGTCGTTCAGGGGCTGCTTTCGGCCTACGAAATGAGTCTCGCCGCGCTGTCACCAGACGGCGACGACAACGGCACCATGCCCCTGCCTATTCACACGCCCAAAGGGATCGTTGCCGGAATGCCTTCCAGGCCATCCATTTCCCATCCACACCCGTCCGCAGCCCCTTACAACGCAGGCGATCCCTCCAAGATGCGGCTGGCAAAGGCCTATAGCACGCACATCTTGCACGTGCTGCACGTGCTGCTCCATGGAAAATGGGATGCCATCTCCATGCTGGACGACGGCGACGATTGGATCACGTCCAAAAGGTTCAACGAATGCGCGTCGCATGCCATTTCTGCGTCGCAGTCCGTCTCGACTATCTTGACTATCGATCCGGAACTGACCTTCATGTCCTATCTCTTCGGGATCTATCTGCTGCAGGGGAGTTTCATTCTTTTGCTCTTCGCGGACAGGATGCCGCAGCTTGGTCCCAACGAGTCGGTTGCGCAGGCGTGTGAGACCATTATTCGAGCTCACGAGGTGTGCGTTGTGACGCTGAGCACAGAATTCCAGAAAAACTTTCGTACCGTTTTGCGATCTACGCTTTATAGTGTTCAAGGTGCAGGAACTACGAATTGGGACGAGCATCGTTCACGACGCAGAGCTTTGTCGCTGTATAGATGGACAAAGGGGGCCAAGGGACTGGCATTGTGA

**Protein sequence:**

MAAEADGQERDRPEPLVGSLAQHSETPDTEHARPRKRTRRACDKCSASRTRCDGECPCRRCEDYGYTCRYNREVKKRGRLPASATANNGNISARRESWPLSTRRGSIRNDSPLSRNANGNSNGSSSNGHAMGYGNAISQASPSSLSATSPSISSSTVAQHPFDDPTDATSARPEGAPLDPRDRVAMSDVRPDVLHSISAPFPQSRPAPPLSVPSLSQVGLQPPSAQRASISNPLSVNVFEGQFGDSEVVVSDESVGYPSPINTRPHAELNRQHRASGKSTHSHERNRESFSTAATTSHDVGGLMLDFFHRAPNEDCWYKFLEPILPYIRNIIPASVACDLLDIFLTDPGSSLFRVASPYILTRVFRKKSIVHPTNPRYTTPALLATILWCVAQTADVMMLHVPGSRAKVVNDLYDLATSLISERDPDRWRRIHGGLRAENEAPHPRLPNAPTMPKTTVNNEPAGEIDDVLTFILLSIAVSGSDFKSDCYKWWSKATRLAFALGLNREDEQCAGPVTPCANPLCSCRKEQDGSLYNLELREERRRVFWLLYALDRHLALSFNSALTIPDSYCEVYSPLPEAIWENLDTISPSEIPARRIGPPTTASGSAFFEYFLPLMAILGDIIEVHHRRRHPRLGGLDDSHSVAVVQGLLSAYEMSLAALSPDGDDNGTMPLPIHTPKGIVAGMPSRPSISHPHPSAAPYNAGDPSKMRLAKAYSTHILHVLHVLLHGKWDAISMLDDGDDWITSKRFNECASHAISASQSVSTILTIDPELTFMSYLFGIYLLQGSFILLLFADRMPQLGPNESVAQACETIIRAHEVCVVTLSTEFQKNFRTVLRSTLYSVQGAGTTNWDEHRSRRRALSLYRWTKGAKGLAL

**2. FDH1 (TRIREDRAFT_60517)**

**Gene sequence, intron in lower case:**

ATGGGTCACTCCAAGTCTGTCCCTCGCTCGTCGTCACAGCCTTCGAGCTCTCGAAACGGCTTCTCTAAACCCTCCCAGAAACCCCAAATGGTCCAGAAAGCGCCCCAGGTCGTGGCGGCGCCGCTGTCATCGCCCGAAGTCCCCAGCGGTCCACTGCCTCTGAGCGGAAAGGTGTTTGCCATCACGGGCGGCGCGAGCGGCATTGGCCGGGCGACTGCTCAGGTACTGTCAAGACGGGGAGCGACTGTCTGCGTCGCCGACGTGGATCACGAAGCGATGAAGGCTGCCGAGGACTATTTCAGCTCCTTTGGGGTGCCGCATATGGTCACCAAGGTGGATGTCTCCAAGCGAAAGGAGGTTGACGCCTGGATCGAGTCGATTATCAAAGAGTATGGCCGGCTGGACGGCGCTGCCAACGTAGCAGGAGTCATTGGAAAGTTTCACGGCGTGTCCCCAGTCTCAGAGCTCGACGACGATGAATGGGATAGGATTATTGGCGTCAACCTGACCGGGACAATGTACTGCATGCGGGCCGAGCTGAGGAATATCGTCGACCGCGGGTCCATTGTCAATGTCTCTTCCATCCACGGCCTCAAGGgtatttttgccgtcttgtttttcccccatcgatatgtgcccgagtgttgtctaatctgttctggccagGCTTTGCAAGGCACGGCGCATACGACGCAAGCAAGCACGGCATCGTCGGCCTCACCCGAGCCGCCGCCCTTGAAAACGGCGAGCGCGAGATCCGCGTCAACAGCGTGGCCCCCGGCGCCATCTACACCCCGCTGATGCAGAAGAACTGGGACTTTTCCGGCCGGCCAAAGGACGCTCCGTTTGACGATCCGACGGCGTTCCAGCGGCAGGGCACGGCGGAGGAGACGGCGAATGTGATTGCGTTTCTGCTGGGGCCCGAGAGCACGTTTGTGAGTGGGAGCGTGTATCGCGTGGACGGAGCCTGGATTTGA

**Coding sequence:**

ATGGGTCACTCCAAGTCTGTCCCTCGCTCGTCGTCACAGCCTTCGAGCTCTCGAAACGGCTTCTCTAAACCCTCCCAGAAACCCCAAATGGTCCAGAAAGCGCCCCAGGTCGTGGCGGCGCCGCTGTCATCGCCCGAAGTCCCCAGCGGTCCACTGCCTCTGAGCGGAAAGGTGTTTGCCATCACGGGCGGCGCGAGCGGCATTGGCCGGGCGACTGCTCAGGTACTGTCAAGACGGGGAGCGACTGTCTGCGTCGCCGACGTGGATCACGAAGCGATGAAGGCTGCCGAGGACTATTTCAGCTCCTTTGGGGTGCCGCATATGGTCACCAAGGTGGATGTCTCCAAGCGAAAGGAGGTTGACGCCTGGATCGAGTCGATTATCAAAGAGTATGGCCGGCTGGACGGCGCTGCCAACGTAGCAGGAGTCATTGGAAAGTTTCACGGCGTGTCCCCAGTCTCAGAGCTCGACGACGATGAATGGGATAGGATTATTGGCGTCAACCTGACCGGGACAATGTACTGCATGCGGGCCGAGCTGAGGAATATCGTCGACCGCGGGTCCATTGTCAATGTCTCTTCCATCCACGGCCTCAAGGGCTTTGCAAGGCACGGCGCATACGACGCAAGCAAGCACGGCATCGTCGGCCTCACCCGAGCCGCCGCCCTTGAAAACGGCGAGCGCGAGATCCGCGTCAACAGCGTGGCCCCCGGCGCCATCTACACCCCGCTGATGCAGAAGAACTGGGACTTTTCCGGCCGGCCAAAGGACGCTCCGTTTGACGATCCGACGGCGTTCCAGCGGCAGGGCACGGCGGAGGAGACGGCGAATGTGATTGCGTTTCTGCTGGGGCCCGAGAGCACGTTTGTGAGTGGGAGCGTGTATCGCGTGGACGGAGCCTGGATTTGA

**Protein sequence:**

MGHSKSVPRSSSQPSSSRNGFSKPSQKPQMVQKAPQVVAAPLSSPEVPSGPLPLSGKVFAITGGASGIGRATAQVLSRRGATVCVADVDHEAMKAAEDYFSSFGVPHMVTKVDVSKRKEVDAWIESIIKEYGRLDGAANVAGVIGKFHGVSPVSELDDDEWDRIIGVNLTGTMYCMRAELRNIVDRGSIVNVSSIHGLKGFARHGAYDASKHGIVGLTRAAALENGEREIRVNSVAPGAIYTPLMQKNWDFSGRPKDAPFDDPTAFQRQGTAEETANVIAFLLGPESTFVSGSVYRVDGAWI

**Codon optimized sequence for expression in *E. coli*:**

ATGGGCCATAGCAAAAGCGTGCCGCGCAGCAGTAGTCAGCCGAGCAGTAGCCGCAACGGCTTTAGCAAACCGAGTCAGAAACCGCAGATGGTTCAGAAAGCCCCGCAAGTGGTGGCGGCCCCGCTGAGCAGCCCGGAAGTGCCGAGCGGCCCGCTGCCGCTGAGCGGCAAAGTGTTCGCGATTACCGGCGGCGCGAGCGGCATTGGCCGTGCGACCGCGCAAGTGTTAAGCCGTCGTGGCGCGACCGTGTGCGTGGCGGATGTGGATCATGAAGCGATGAAAGCGGCGGAAGATTATTTTAGCAGCTTTGGCGTGCCGCATATGGTGACCAAAGTGGATGTGAGCAAACGCAAAGAAGTGGATGCGTGGATTGAAAGCATTATTAAAGAATATGGCCGCCTGGATGGCGCGGCGAACGTGGCGGGCGTGATTGGCAAATTTCATGGCGTGAGCCCGGTGAGCGAACTGGATGACGATGAATGGGATCGCATTATTGGCGTGAACCTGACCGGCACCATGTATTGCATGCGCGCGGAACTGCGCAACATTGTGGATCGCGGCAGCATTGTGAACGTGAGCAGCATTCATGGCCTGAAAGGCTTTGCGCGCCATGGCGCGTATGATGCGAGCAAACATGGCATTGTGGGCCTGACCCGCGCGGCCGCGCTGGAAAACGGCGAACGCGAAATTCGCGTGAACAGCGTGGCGCCGGGCGCGATTTATACCCCGCTGATGCAGAAAAACTGGGATTTTAGCGGCCGCCCGAAAGATGCGCCGTTTGATGATCCGACCGCGTTTCAGCGCCAAGGCACCGCGGAAGAAACCGCGAACGTGATTGCGTTTCTGCTGGGCCCGGAAAGCACCTTTGTGAGCGGCAGCGTGTATCGCGTGGATGGCGCGTGGATTTAA

**3. Afc95A (TRIREDRAFT_5807)**

**Gene sequence, introns in lower case:**

ATGGTCACAATGTTGCCAGCAAGGCTCGTTGTTGTCGCGACTTGCGCCCTGGGCGTAGGGGCAAGGAAACTATGGGCAACTGAACCAGCCGATGCAGGGAACATTATCATGACGGCATATCCGCTAGGAAATGGAAAACTTGGAGgtgagtctgtgaacaacgctgcccaatctgaaggcaaatatctaatttggtatatatagCAATGCCGCTTGGGGTGGTAGGGGAGGATATCGTGGTACTCAACGAACATAGTCTTTGGGCCGGAGGCCCTTTCCAGAGTCCGgtaagccctcaaccctagtactttcaactgacagcaacatttgctcaaagacctgttcttgactctgctagGATTACATTGGCGGCAACCCACCGGCTCCGGTCTATACGGCCCTTCCCGGCATTAGAGAGACGATTTGGAAGACTCAAATCAACAATGgttcgtttcacctgtcttgagtcacttcattctgttctgacgcccatttgccctcgagcagACATCAGTGCTCTGTATGGGGATCCTGCATACTACTATTATGGCAACTACGAGACCCTAGGCAATCTCACCGTCAACATTGCGGGAGTCAGCAAATACACATCTTATAACCGCGCGCTGGATCTTGAAACTGGCATTCACACCACCGAGTTCAAGGCAAACGGGGCCAAGTTCACCATgtgagtaccttacgctgaaagagcgctttgtactcccgcagaaacccgtcacggaagttgacccaaaacagAACTACCTTTTGCACGTTCCCCGACCAAGTCTGCGCTTACAACATCCAGTCCAGCAAGCCGCTCCCTGCTGTGACAATTGGACTGCGGGACTCTTTACGAAGCAACCCAGCGTCCAACCTGACTTGCGACGCGAATGGAGTGCATTTGAGAGGCCAAACCCAGCAGGATATTGGCATGATCTTTGATGCCCGCGCCCAACTCATCAACCGACCTAAGCGAGCGACGTGCACGTCATCTCATGGTCTCTCCGTTCCGTCGGACGGCAGAACGACTTCTCTCACTGTCGTGTACGCTGCTGGGACCAACTATGATCAGAAGAAGGGAACCAAGGCGAGCAACTACTCTTTCAAGGGCGTCGACCCAGCGCCAGCTGTTCTCTCGACGATTAAGAAGGTCTCCCAAAAGAGCTTCAATAGCATGTATAATGCGCATATAAAGGACCACAATGGTTTGTTCAGCCAATTCAGCCTGGACCTCCCAGACCCCGAGAAGTCGGCTTCCGTGCCGACGGCGACGCTGATGGAAAATTACGACTACGACCTCGGCGACCCATTTGTTGAGAATCTCCTCTTCGACTATGGAAGGTACCTTTTCATCGGCTCCTGCAGAGATGGGTCGCTGCCTCCCAATCTGCAGGGTATTTGGACGGAATCGCTCACTCCGGCCTGGAGCGCAGACTATCACGTCGATGTGAACGTCCAGATgtacgtttgacactttgcgccccagatccgactccatttaaccatgatataatcttagGAACCACTGGCACACTGAGCAAACTGGCCTTGGAGAGATTCAGGGCCCCCTGTGGGACTTCATCATCGATACATGGGTGCCTCGAGGAACAGAAACAGCTGCATTGCTGTATGACGCCCCAGGATTTGTTGGATTCAGCAACCTCAACACATTCGGTTTCACGGGgtaagctggacatattccggacgaggttgagcagatttgctgacgagttgaagCCAAATGAACGCCGCTGTGTGGTCCAACTACCCAGCCTCTGCTGCTTGGCTGAgtgagtgaatttgtatagactctctctccagcctgtccgtatattgacatcatgattcaacagTGCAAAACGTCTGGAACCGATACGACTACAGCCGCGATACGCACTGGTGGAAGACGGTCGGATATCCTCTCATGAAATCCATTGCCGAGTACTGGATCCACGAGATGGTGCCCGATCTGTATTCCAACGACGGCACCCTCGTCGCGGCTCCTTGCAACTCACCGGAGCACGGCTGGACGgtaagatactcggttaaagctcatttttgaggcatgatgctgaattcaggccagACATTCGGCTGCACACACTACCAGCAGCTCGTGTGGGAAGTTTTTGACCATGTGATCGAGGGCTGGGAAGCCTCAGGCGATAAGAACACCACGTTCCTCGAGACCGTCAAGGAGACCCAGTCGAAGCTGTCTCCAGGAATCATCATTGGCTGGTTTGGTCAAATCCAAGgtacgcatacggccgcgacggccacatatgagatcaccttgctaagtcagacacagAATGGAAAATTGGTTGGGATCAACCCAACGACGAGCATCGCCACCTTTCCCACCTCGTCGGCTGGTATCCCGGCTACAGCATCGGCACACACATGTGGAATAAGACCGTCACAGACGCCGTCAATGTCAGTTTGACAGCCCGAGGCAACGGCACGGCAGACTCAAACACCGGCTGGGAGAAAGTCTGGCGAGTCGCCTGCTGGGCGCAGCTCAACAACACTGACATCGCGTACACATACCTCAAATATGCCATTGACATGAACTACGCAAACAACGGCTTCTCCGTCTACACCACAGGCAGCTGGCCATACGAGCTCGCAGCGCCCTTCCAGATCGACGCCAACTTTGGATACAGCGCTGCCGTGCTGGCGATGCTCATCACCGACCTGCCAGTTCCGTCTGCGTCCAAGGCTATCCACACCGTCATCTTGGGGCCGGCCATTCCGCCAGAGTGGAAGGGCGGCTCTGTTCGGGGCATGCGTATCAGAGGCGGAGGATCCGTCGACTTTTCGTGGGACGATAATGGCTTGGTAAATAAGGCGAAGCTGCACAACCATAAGGAGGCGATTAAGATCGTCGATGTGAACGGCAAGGTCTTGATTCATCAGTGA

**Coding sequence:**

ATGGTCACAATGTTGCCAGCAAGGCTCGTTGTTGTCGCGACTTGCGCCCTGGGCGTAGGGGCAAGGAAACTATGGGCAACTGAACCAGCCGATGCAGGGAACATTATCATGACGGCATATCCGCTAGGAAATGGAAAACTTGGAGCAATGCCGCTTGGGGTGGTAGGGGAGGATATCGTGGTACTCAACGAACATAGTCTTTGGGCCGGAGGCCCTTTCCAGAGTCCGGATTACATTGGCGGCAACCCACCGGCTCCGGTCTATACGGCCCTTCCCGGCATTAGAGAGACGATTTGGAAGACTCAAATCAACAATGACATCAGTGCTCTGTATGGGGATCCTGCATACTACTATTATGGCAACTACGAGACCCTAGGCAATCTCACCGTCAACATTGCGGGAGTCAGCAAATACACATCTTATAACCGCGCGCTGGATCTTGAAACTGGCATTCACACCACCGAGTTCAAGGCAAACGGGGCCAAGTTCACCATAACTACCTTTTGCACGTTCCCCGACCAAGTCTGCGCTTACAACATCCAGTCCAGCAAGCCGCTCCCTGCTGTGACAATTGGACTGCGGGACTCTTTACGAAGCAACCCAGCGTCCAACCTGACTTGCGACGCGAATGGAGTGCATTTGAGAGGCCAAACCCAGCAGGATATTGGCATGATCTTTGATGCCCGCGCCCAACTCATCAACCGACCTAAGCGAGCGACGTGCACGTCATCTCATGGTCTCTCCGTTCCGTCGGACGGCAGAACGACTTCTCTCACTGTCGTGTACGCTGCTGGGACCAACTATGATCAGAAGAAGGGAACCAAGGCGAGCAACTACTCTTTCAAGGGCGTCGACCCAGCGCCAGCTGTTCTCTCGACGATTAAGAAGGTCTCCCAAAAGAGCTTCAATAGCATGTATAATGCGCATATAAAGGACCACAATGGTTTGTTCAGCCAATTCAGCCTGGACCTCCCAGACCCCGAGAAGTCGGCTTCCGTGCCGACGGCGACGCTGATGGAAAATTACGACTACGACCTCGGCGACCCATTTGTTGAGAATCTCCTCTTCGACTATGGAAGGTACCTTTTCATCGGCTCCTGCAGAGATGGGTCGCTGCCTCCCAATCTGCAGGGTATTTGGACGGAATCGCTCACTCCGGCCTGGAGCGCAGACTATCACGTCGATGTGAACGTCCAGATGAACCACTGGCACACTGAGCAAACTGGCCTTGGAGAGATTCAGGGCCCCCTGTGGGACTTCATCATCGATACATGGGTGCCTCGAGGAACAGAAACAGCTGCATTGCTGTATGACGCCCCAGGATTTGTTGGATTCAGCAACCTCAACACATTCGGTTTCACGGGCCAAATGAACGCCGCTGTGTGGTCCAACTACCCAGCCTCTGCTGCTTGGCTGATGCAAAACGTCTGGAACCGATACGACTACAGCCGCGATACGCACTGGTGGAAGACGGTCGGATATCCTCTCATGAAATCCATTGCCGAGTACTGGATCCACGAGATGGTGCCCGATCTGTATTCCAACGACGGCACCCTCGTCGCGGCTCCTTGCAACTCACCGGAGCACGGCTGGACGACATTCGGCTGCACACACTACCAGCAGCTCGTGTGGGAAGTTTTTGACCATGTGATCGAGGGCTGGGAAGCCTCAGGCGATAAGAACACCACGTTCCTCGAGACCGTCAAGGAGACCCAGTCGAAGCTGTCTCCAGGAATCATCATTGGCTGGTTTGGTCAAATCCAAGAATGGAAAATTGGTTGGGATCAACCCAACGACGAGCATCGCCACCTTTCCCACCTCGTCGGCTGGTATCCCGGCTACAGCATCGGCACACACATGTGGAATAAGACCGTCACAGACGCCGTCAATGTCAGTTTGACAGCCCGAGGCAACGGCACGGCAGACTCAAACACCGGCTGGGAGAAAGTCTGGCGAGTCGCCTGCTGGGCGCAGCTCAACAACACTGACATCGCGTACACATACCTCAAATATGCCATTGACATGAACTACGCAAACAACGGCTTCTCCGTCTACACCACAGGCAGCTGGCCATACGAGCTCGCAGCGCCCTTCCAGATCGACGCCAACTTTGGATACAGCGCTGCCGTGCTGGCGATGCTCATCACCGACCTGCCAGTTCCGTCTGCGTCCAAGGCTATCCACACCGTCATCTTGGGGCCGGCCATTCCGCCAGAGTGGAAGGGCGGCTCTGTTCGGGGCATGCGTATCAGAGGCGGAGGATCCGTCGACTTTTCGTGGGACGATAATGGCTTGGTAAATAAGGCGAAGCTGCACAACCATAAGGAGGCGATTAAGATCGTCGATGTGAACGGCAAGGTCTTGATTCATCAGTGA

**Protein sequence, predicted signal peptide underlined:**

MVTMLPARLVVVATCALGVGARKLWATEPADAGNIIMTAYPLGNGKLGAMPLGVVGEDIVVLNEHSLWAGGPFQSPDYIGGNPPAPVYTALPGIRETIWKTQINNDISALYGDPAYYYYGNYETLGNLTVNIAGVSKYTSYNRALDLETGIHTTEFKANGAKFTITTFCTFPDQVCAYNIQSSKPLPAVTIGLRDSLRSNPASNLTCDANGVHLRGQTQQDIGMIFDARAQLINRPKRATCTSSHGLSVPSDGRTTSLTVVYAAGTNYDQKKGTKASNYSFKGVDPAPAVLSTIKKVSQKSFNSMYNAHIKDHNGLFSQFSLDLPDPEKSASVPTATLMENYDYDLGDPFVENLLFDYGRYLFIGSCRDGSLPPNLQGIWTESLTPAWSADYHVDVNVQMNHWHTEQTGLGEIQGPLWDFIIDTWVPRGTETAALLYDAPGFVGFSNLNTFGFTGQMNAAVWSNYPASAAWLMQNVWNRYDYSRDTHWWKTVGYPLMKSIAEYWIHEMVPDLYSNDGTLVAAPCNSPEHGWTTFGCTHYQQLVWEVFDHVIEGWEASGDKNTTFLETVKETQSKLSPGIIIGWFGQIQEWKIGWDQPNDEHRHLSHLVGWYPGYSIGTHMWNKTVTDAVNVSLTARGNGTADSNTGWEKVWRVACWAQLNNTDIAYTYLKYAIDMNYANNGFSVYTTGSWPYELAAPFQIDANFGYSAAVLAMLITDLPVPSASKAIHTVILGPAIPPEWKGGSVRGMRIRGGGSVDFSWDDNGLVNKAKLHNHKEAIKIVDVNGKVLIHQ
